# Lipid droplet-associated proteins integrate lipid metabolism, cell cycle, and cytoskeletal remodeling in colon cancer

**DOI:** 10.1016/j.jlr.2026.101165

**Published:** 2026-10-06

**Authors:** Patricia Snarski, Abigail Drago, Michele Daniels, Muhammad Anas Naeem, Angelle N. King, Emmanuelle Ruiz, Cera Webb, Rithvik Vutukuri, Tyler Rorison, Suzana D. Savkovic

**Affiliations:** Department of Pathology and Laboratory Medicine, Tulane University School of Medicine, New Orleans, USA

**Keywords:** lipid droplets, lipid metabolism, cell cycle, cytoskeleton remodeling, colon cancer

## Abstract

Lipid remodeling is a hallmark of colonic tumorigenesis, with lipid droplets emerging as key drivers of cellular metabolism and tumor growth. This study investigates how dysregulated lipid droplet-associated proteins facilitate human colon cancer growth. Using a publicly available, highly selective lipid droplet proteomics dataset, we generated a transcriptomic signature representing 151 lipid droplet-associated proteins. This signature was significantly enriched in human colon cancer transcriptomes, distinguishing tumor from normal samples. Representative proteins from this signature were dysregulated in human colonic tumors. Regulators of lipid metabolism, acyl-CoA synthetase (ACSL) 3/4, hypoxia-inducible lipid droplet-associated protein, and phospholipase domain-containing protein 3, had increased protein levels in colon tumors, corresponding to upregulated transcripts, predicting poor patient survival. Protein levels of cell cycle regulators Cell Division Cycle 42, Proline Rich 11, Stratifin, and TAT-interacting Protein, as well as cytoskeletal regulators desmoplakin, Molecule Interacting with CasL 2, LIM and SH3 protein 1, and Cofilin 1, were increased despite unchanged or reduced transcript levels in colonic tumors. Moreover, oleic acid stimulated lipid droplet accumulation increased RNA and protein levels of these regulators in colon cancer HCT116 and HT29 cells, with ACSL3, Cell Division Cycle 42, Stratifin, and TAT-interacting Protein showing cell line-specific differences in expression. Functional studies demonstrated silencing ACSL4, Proline Rich 11, LIM and SH3 protein 1, or Molecule Interacting with CasL 2 attenuated proliferation, cell cycle progression, and migration of colon cancer cells stimulated with oleic acid. These findings suggest lipid droplets as a hub for metabolic and tumorigenic regulators highlighting their associated proteins as potential biomarkers and therapeutic targets.

Colon cancer, the second leading cause of cancer-related deaths worldwide, is driven by oncogenic signaling and dysregulated metabolism 1, 2. Aberrant lipid metabolism in colon cancer cells and the surrounding tumor microenvironment is recognized as a hallmark of colon cancer progression 3, 4. Emerging findings reveal that colonic tumors contain a marked accumulation of lipid droplets, intracellular lipid-storing organelles 5, 6, 7, though the mechanisms underlying their tumor-promoting role are not fully elucidated.

Lipid droplets were historically viewed as passive lipid storage depots predominantly found in fat-storing adipose and liver tissue 8, 9. Recent findings have demonstrated that lipid droplets, composed primarily of triglycerides and proteins embedded in their phospholipid monolayer, are dynamic organelles critical for diverse cellular functions 8, 9. Our lab and others have demonstrated increased lipid droplet accumulation in non-fat-storing tissues, such as colon, driving tumorigenesis 5, 7, 10, 11, 12, 13, 14. These organelles may promote tumorigenesis by providing building blocks and metabolic energy while regulating cell cycle and cytoskeletal dynamics 5, 15. We have previously demonstrated that several lipid droplet-associated proteins, including Diacylglycerol O-acyltransferase 1/2 (DGAT1/2) and adipose triglyceride lipase (ATGL), are significantly increased in human colonic tumors 11, 12, 13. DGAT1/2 catalyze the final step in triacylglycerol synthesis and promote lipid droplet biogenesis 9, 14, 16. ATGL, the rate-limiting enzyme in triacylglycerol lipolysis, is critical for lipid droplet utilization 17, 18. Targeting DGAT1/2 or ATGL attenuates tumorigenic signaling and colon cancer cell growth, predicting improved patient survival 12, 13. These findings underscore the need to characterize the mechanisms by which other lipid droplet-associated proteins contribute to colonic tumor growth.

Bersuker *et al.* generated and validated a comprehensive lipid droplet proteome using a proximity-labeling approach, identifying numerous lipid droplet-associated proteins (19). While these proteins are involved in diverse biological processes, their functional significance and dependence on lipid droplets in colonic tumorigenesis remain unexamined. Here, we determined whether these lipid droplet-associated proteins are dysregulated in human colon cancer, their role in promoting tumor growth, and evaluated their potential as novel biomarkers and therapeutic targets.

## Materials and methods

### Human colonic samples

Human colon tumor tissues with matched adjacent normal tissues were obtained as a tissue microarray (C0321a, TissueArray.com, Derwood, MD, n = 16 patients). Transcriptomic data were obtained from The Cancer Genome Atlas (TCGA) colon cancer cohort (TCGA; https://portal.gdc.cancer.gov/projects/TCGA-COAD), which included 461 tumors and 41 adjacent normal samples. This dataset was also assessed by the Human Protein Atlas (https://www.proteinatlas.org) and through the TIMER3 (https://compbio.cn/timer3/) platform 20, 21.

### Cell culture

Human colon cancer cells, HT29 and HCT116, obtained from the ATCC(Manassas, VA), were propagated in McCoy’s 5A media (Sigma-Aldrich, St. Louis, MO) containing 10% FBS (Peak Serum, Wellington, CO) and maintained under standard culture conditions (37°C with 5% CO_2_). These cell lines have been authenticated by ATCC through DNA profiling and were tested for mycoplasma contamination.

### Treatments

Human colon cancer cells, serum starved overnight, were treated with oleic acid (300 μM, Sigma-Aldrich, O3008) conjugated to BSA as a carrier protein. Oleic acid treatments were performed for 6 or 24 h. In parallel, control cells were maintained in serum free media.

### siRNA transfection

Colon cancer cells were diluted to a concentration of 5 × 10^6^ cells/ml in Gene Pulser Electroporation Buffer (Bio-Rad, 1652677), 400 μl of this suspension was then transferred to a 0.4 cm electroporation cuvette (Bio-Rad, 1652081). Cells were then transfected with siRNA targeting ACSL4, Proline Rich 11 (PRR11), LIM and SH3 protein 1 (LASP1), Molecule Interacting with CasL 2 (MICAL2) (20 nM, Dharmacon J-009364-05-0005, J-007019-19-0005, J-010519-10-0005, J-010189-05-0005), non-targeting scrambled siRNA control (20 nM, Dharmacon D-001810-10-05), or electroporation buffer alone (negative control). Electroporation was performed using a Bio Rad Gene Pulser (two pulses, 10 ms each, 250 V). Cells were allowed to recover for 24 h following transfection before subsequent treatment or sample collection.

### Immunohistochemistry

Immunohistostaining was performed on human colonic tumors and matched normal tissue from a tissue microarray, as described 13, 22. Primary antibodies and their dilutions used were as follows: ACSL3 (Thermo Fisher Scientific, PA5-82427, 1:500), ACSL4 (Thermo Fisher Scientific, PA5-27137, 1:500), hypoxia-inducible lipid droplet-associated protein (HILPDA) (Novus, NBP3-03847, 1:100), phospholipase domain-containing protein 3 (PNPLA3) (Cell Signaling, 95546, 1:100), Cell Division Cycle 42 (CDC42) (Abcam, ab187643, 1:100), PRR11 (Thermo Fisher Scientific, MA5-26459, 1:100), Stratifin (SFN) (Thermo Fisher Scientific, PA5-95056, 1:500), TAT-interacting Protein (TIP30) (Abcam, ab177961, 1:500), DSP (Proteintech, 25318-1-AP, 1:2000), MICAL2 (Abcam, ab238901, 1:200), LASP1 (Proteintech, 10515-1-AP, 1:100), Cofilin 1 (CFL1) (Thermo Fisher Scientific, MA5-17275, 1:500). Immunohistostaining procedures were performed simultaneously in the Nemesis autostainer (Biocare Medical) to maintain uniform conditions. Tissue sections were incubated with primary antibodies and washed, followed by HRP-Polymer secondary antibodies (Biocare Medical, M4U534), except for PNPLA3, which was incubated with Goat on Rodent AP Polymer (Biocare Medical, GAP514). Sections were treated with Betazoid 3,3′-diaminobenzidine (DAB) chromogen (Biocare Medical, BDB2004) and counterstained with Cat hematoxylin (Biocare Medical, CATHEM). Slides were scanned using the Akoya Slide Scanner (Akoya Biosciences). Color deconvolution was used to separate the hematoxylin and DAB staining to quantify the protein of interest 13, 22, 23. Four representative images were captured from each tissue type and DAB-positive staining was quantified in multiple regions (n = 4–15) using ImageJ.

### Immunofluorescence

Experimental colon cancer cells were fixed with 3.7% paraformaldehyde, permeabilized with 0.1% Triton X-100 in PBS for 10 min at room temperature, and immunostained using the following primary antibodies: ACSL3 (Thermo Fisher Scientific, PA5-29507, 1:500), ACSL4 (Thermo Fisher Scientific, PA5-27137, 1:250), HILPDA (Bioss, BS-15488R, 1:250), PNPLA3 (Thermo Fisher Scientific, PA5-18901, 8 μg/ml), PRR11 (Abcam, ab237526, 1:100), CDC42 (Abcam, ab155940, 1:60), SFN (Abcam, ab151504, 1:500), TIP30 (Thermo Fisher Scientific, PA5-27109, 1:500), MICAL2 (Proteintech, 13965-1-AP, 1:400), LASP1 (Thermo Fisher Scientific, PA5-52907, 0.75 μg/ml), CFL1 (Thermo Fisher Scientific, PA5-27627, 1:450), and DSP (Proteintech, 25318-1-AP, 1:1125). Following primary antibody incubation, cells were washed and incubated for 1 h at room temperature in the dark with Alexa Fluor 594-conjugated goat anti-rabbit IgG (H + L) (2 μg/ml, Invitrogen, A-11012) or donkey anti-goat IgG (H + L) (2 μg/ml, Invitrogen, A-11058) for the PNPLA3 primary antibody. Lipid droplets were visualized by staining with BODIPY™ 493/503 (5 μM, Invitrogen, D3922), and nuclei were counterstained with 4',6-diamidino-2-phenylindole (DAPI) (1 μg/ml, Thermo Scientific Scientific, 62248). High-resolution fluorescence images were acquired using Cytation 5 Cell Imaging system (Agilent BioTek) and analyzed using CellSens software (Olympus). Manual intensity thresholds were established separately for each protein channel and applied consistently across all treatment groups, whereas a single, uniform threshold was used for the BODIPY channel across all images. Protein-positive area and BODIPY-positive area were quantified and normalized to the DAPI-positive area.

### Immunoblot

Cells were lysed in RIPA Lysis and Extraction Buffer (Thermo Fisher Scientific, 89901) supplemented with Halt Protease and Phosphatase Inhibitor cocktail (Thermo Fisher Scientific, 78442). Lysates were centrifuged at 14,000g for 15 min at 4°C, and supernatants were collected. Protein concentrations were determined using the Pierce BCA Protein Assay Kit (Thermo Fisher Scientific, A55864). Equal amounts of protein were resolved by SDS-PAGE and transferred onto nitrocellulose membranes (0.2 μm; Bio-Rad, 1620112). Membranes were blocked for 1 h at room temperature in Intercept Blocking Buffer (LI-COR Biosciences, 927-60001) and incubated overnight at 4°C with the following primary antibodies: ACSL4/FACL4 (Proteintech, 22401-1-AP, 1:1000); PRR11 (Thermo Fisher Scientific, PA5-113175, 1:1000); LASP1 (Invitrogen, PA5-52907, 1:1000); MICAL2 (Proteintech, 13965-1-AP, 1:1000); β-actin (loading control; Cell Signaling Technology, 4970, 1:5000). Following three washes in TBST (TBS+0.1% Tween-20), membranes were incubated for 1 h at room temperature with secondary antibodies (ACSL4, PRR11, LASP1, MICAL2: Goat anti-Rabbit, LI-COR IRDye680RD, 1:5000; β-actin: Goat anti-Mouse LI-COR IRDye800CW, 1:5000). Blots were visualized on a Chemidoc MP system (Bio-Rad). Densitometric quantification was performed using ImageLab software (Bio-Rad).

### Proliferation assay

Cell proliferation was assessed by BrdU incorporation. Experimental colon cancer cells were treated with BrdU (10 μM, Thermo Fisher Scientific, B23151) for 3 h, followed by fixation with 3.7% paraformaldehyde and immunostaining with anti-BrdU antibody (Cell Signaling Technology, 5292, 1:1000) and Alexa Fluor™ 594-conjugated goat anti-mouse IgG (H + L) secondary antibody, as previously described (13). High-resolution fluorescent images were acquired using a Cytation 5 Cell Imaging System (Agilent BioTek) and analyzed using CellSens software (Olympus). BrdU-positive nuclei were quantified and expressed as a percentage of the total DAPI-positive nuclei.

### Flow cytometry

Cell cycle was assessed via flow cytometry (BD FACS ARIA III) as we described before (12). Briefly, human colon cancer cells, transfected and serum starved for three days, were stained for DNA (Propidium Iodide). The experiment was performed four independent times, and data were analyzed using Floreada software (https://floreada.io).

### Migration assay

Cell migration was assessed using a scratch assay. Experimental colon cancer cells were grown to a confluent monolayer, and a uniform scratch was generated using a sterile pipette tip. Following the treatments, cells were monitored for 24 h. Brightfield images were acquired immediately after scratch formation (0 h) and every 6 h thereafter (6, 12, 18, and 24 h, BioSpa-Cytation 5, Gen5 3.10 Imager, CellSens software). Migration was calculated as the area of the scratch at 0 h (A_*0h*_) subtracted from the area of the scratch at the time of interest (A_*t*_), normalized to A_*0h*_ times 100 ([(A_*0h*_ – A_*t*_)/A_0*h*_] x 100).

### Quantitative PCR

Total RNA was extracted from experimental cells using RNeasy Lipid Tissue Mini Kit (Qiagen, 74,804) according to the manufacturer’s protocol. Total RNA isolation and complementary cDNA synthesis were performed as previously described (13). Primers (Thermo Fisher Scientific) used in this study were as follows; hHILPDA F 5′-GCTTTTGTCTCCGGGTCCA-3′ R 5′-GCTAGTTGGCTTCTGGTGGT-3′; hLASP1 F 5′- CTTCGCCTCAAGCAACAGAGTG-3′ R 5′ TGTCTGCCACTACGCTGAAACC-3′; hCDC42 F 5′- AACGCCCCGGTGGAGAA-3′ R 5′-TGTCATAATCCTCTTGCCCTGC-3′; hPRR11 F 5′- CGTGGGAGAGGCCACAG-3′ R 5′-TTGAACTTGGGCATGATTTTAGTCT-3′; hTIP30 F 5′- AGCCCTTGTTCCTCGGGATA-3′ R 5′- ACGAACAAATCCCTCCGCC-3′; hACSL3 F 5′- TGACACAAGGGCGCATATCT-3′ R 5′- CCCAACAACGAATTCTGTATCAGT-3′; hDSP F 5′-CAAAAGCAGGCTTTAGAGGCAT-3′ R 5′-TTACGCCTGCACCTCTCTTC-3′; hMICAL2 F 5′-AGCAACAAAGAGAGGAGGAGG-3′ R 5′-GTCAGCCAAGAAGTGGGTGT-3′; hACSL4 F 5′-TCCTTTTTGCGAGCTTTCCG-3′ R 5′- TCTGTGATTTCAATCGGAGGGG-3′; hPNPLA3 F 5′-GAGCCTAAGACGATCTCCTGC-3′ R 5′- CAGTGACAACCATCCCAGCA-3′; hSFN F 5′- TCCACTACGAGATCGCCAACAG-3′ R 5′- GTGTCAGGTTGTCTCGCAGCA-3′; hB2M F 5′-AGGCTATCCAGCGTACTCCA-3′ R 5′-TCATCCAATCCAAATGCGGC-3′. Relative mRNA levels were determined using the comparative Ct (ΔΔCt) method with Beta-2 Microglobulin as a housekeeping control. Quantitative PCR reactions were performed using the CFX Opus 96 Real Time PCR System (Bio-Rad) with SYBR Green qPCR Master Mix (Thermo Fisher Scientific, A59529).

### Transcriptome analysis

The lipid droplet transcriptomic signature was generated by converting the published lipid droplet proteome into a curated gene set through protein-to-gene mapping, annotation, and identifier harmonization using UniProt (24), yielding a nonredundant gene expression signature for downstream transcriptomic analyses. Unsupervised hierarchical clustering was performed using a Spearman correlation-based distance metric to visualize expression patterns of the 151 lipid droplet-associated genes (LDs_151_) across TCGA colonic tumors and adjacent normal samples. Genes were filtered using a threshold of ≥1.5-fold change relative to control and a false discovery rate < 0.05, using analytical platforms described previously 13, 22, 23. Principal component analysis was performed to estimate the heterogeneity of colon samples using 151 LD genes. The values of the first principal component, presenting the highest variance, were further plotted on a boxplot to visualize the difference between tumor and normal samples, explained by the lipid droplet-associated genes. Moreover, gene expression patterns across the Consensus Molecular Subtypes (CMS) were analyzed and visualized using the CMScaller package in R software.

### Statistics

All experiments were conducted with a minimum of three independent biological replicates per group, and investigators were blinded to group allocation. Data were reported as mean ± standard deviation. Statistical analysis was performed by Student’s unpaired *t* test or ANOVA (one or two-way) with Tukey’s multiple comparisons (GraphPad Prism version 10.0). A *P*-value <0.05 was considered significant, and specific statistical tests are described in figure legends.

## Results

### Transcriptional signature representing LDs_151_ is enriched in human colon cancer transcriptomes

To determine whether proteins associated with lipid droplets are dysregulated in human colon cancer, we generated a lipid droplet transcriptomic signature by converting a published lipid droplet proteome (19) into a gene set through protein-to-gene mapping and annotation using bioinformatic approaches (24). This signature comprised 151 transcripts (LDs_151_; supplemental Table S1) representing regulators of diverse cellular functions, including lipid metabolism, cell cycle, and cytoskeletal organization. Furthermore, we evaluated the enrichment of this LDs_151_ signature in publicly available TCGA colon cancer cohorts. Using unsupervised hierarchical clustering and principal component analysis, the LDs_151_ signature significantly distinguished colon cancer samples from normal tissue in a publicly available transcriptomic dataset (TCGA, Fig. 1A, B). This LDs_151_ signature provides a framework to investigate how distinct classes of lipid droplet-associated proteins may contribute to tumorigenesis.

**Fig. 1:**
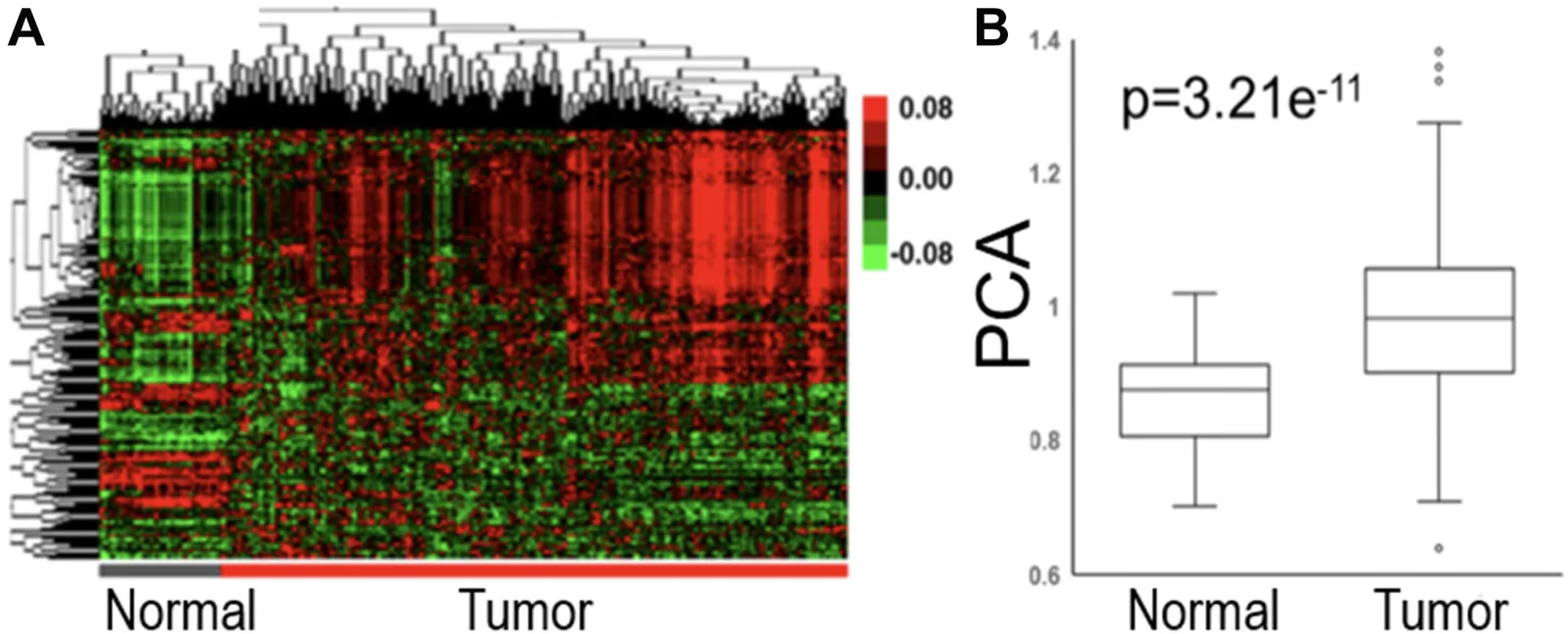
A and B: Enrichment of lipid droplet transcriptional signature in human colon cancer transcriptomes. A publicly available, highly selective lipid droplet proteomics dataset served as a basis for protein-to-gene mapping and annotation to develop a transcriptomic signature representing 151 lipid droplet-associated proteins (LDs_151_). Unsupervised hierarchical clustering (heatmap) and principal component analysis (PCA) of LDs_151_ signature separated publicly available human colonic tumors from normal samples (TCGA, n = 461 tumors and n = 41 normal, *P* = 3.2e^−11^). TCGA, The Cancer Genome Atlas.

### Lipid droplet-associated regulators of lipid metabolism are elevated in human colon cancer

We first assessed lipid droplet abundance in human colonic tumor tissues by examining the level of the well-established lipid droplet marker, Perilipin 2 10, 15, 17. Immunohistostaining demonstrated a significant increase in Perilipin 2 levels in human colon tumor samples compared with matched adjacent normal tissues (supplemental Fig. S1). Next, we examined the levels of selected lipid droplet-associated proteins regulating lipid metabolism in these colonic tumor samples. Immunohistostaining revealed significantly increased protein levels of acyl-CoA synthetase 3 and 4 (ACSL3 and ACSL4), HILPDA, and patatin-like PNPLA3 in human colonic tumors (Fig. 2A). Furthermore, transcriptomic analysis of a publicly available dataset showed significantly elevated expression of these genes in tumors compared with normal samples (Fig. 2B). In addition, elevated transcript levels of *ACSL3* and *ACSL4*, which convert free long-chain fatty acids into acyl-CoA esters (25), were associated with poor survival in colon cancer patients (Fig. 2C). Similarly, increased transcript levels of *HILPDA*, a promoter of lipid storage within lipid droplets (25), showed a trend toward poorer patient survival outcomes (Fig. 2C), whereas increased *PNPLA3*, a regulator of lipid droplet growth and utilization (26), was unrelated to patient survival (Fig. 2C). These findings demonstrate that several lipid droplet-associated proteins regulating lipid metabolism are upregulated in human colon cancer, suggesting that alterations in protein composition of lipid droplets may contribute to colonic tumor progression.

**Fig. 2:**
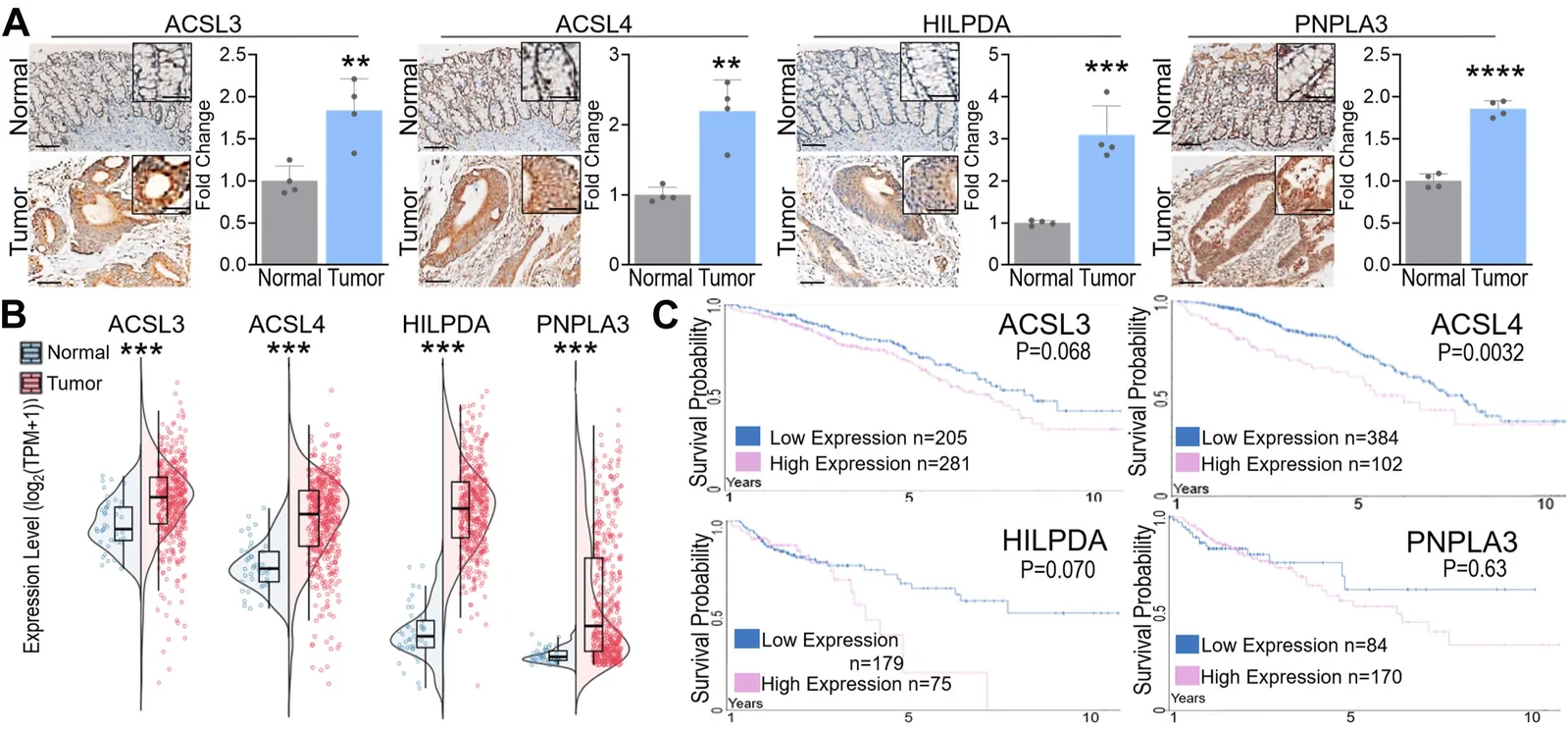
A: Lipid droplet-associated proteins regulating lipid metabolism, ACSL3, ACSL4, HILPDA, and PNPLA3, in human colonic tumors. Immunohistostaining of Stage 2 human colon cancer tumor tissue and adjacent normal tissue (the scale bar represents 50 μm; inset 20 μm). Graphs represent densitometric analysis using ImageJ. Data are presented as mean ± SD (n = 4 patients, n = 4 average images per patient). Statistical significance was determined by unpaired *t* test; *P* < 0.05 was considered statistically significant (∗∗*P* < 0.01, ∗∗∗*P* < 0.001, ∗∗∗∗*P* < 0.0001 versus normal). B: Violin plots of *ACSL3*, *ACSL4*, *HILPDA*, and *PNPLA3* transcripts in publicly available transcriptomes of colon cancer patients (TCGA via TIMER3, n = 478 tumor, n = 41 normal, ∗∗∗*P* < 0.001 versus normal). C: Kaplan-Meier survival probability of *ACSL3*, *ACSL4*, *HILPDA*, and *PNPLA3* in colon cancer transcriptomes (Human Protein Atlas validated TCGA dataset, *ACSL3* n = 205 low expression and n = 281 high expression, total n = 486; *ACSL4* n = 384 low expression and n = 102 high expression, total n = 486; *HILPDA* n = 179 low expression and n = 75 high expression, total n = 254; *PNPLA3* n = 84 low expression and n = 170 high expression, total n = 254). ACSL, acyl-CoA synthetase; HILPDA, hypoxia-inducible lipid droplet-associated protein; PNPLA3, phospholipase domain-containing protein 3; TCGA, The Cancer Genome Atlas.

### Lipid droplet-associated cell cycle regulators are altered in human colon cancer

Several cell cycle regulators associated with lipid droplets, such as PRR11, CDC42, SFN, and 30 kDa HIV-1 TIP30, were further examined in human colon cancer (immunohistostaining, TCGA). PRR11, which promotes progression from S into G2/M phase (27), had elevated protein and transcript levels in colonic tumors and was associated with poorer patient survival (Fig. 3A–C). Furthermore, dysregulated protein and transcript patterns in human colon cancer were seen in several other cell cycle regulators associated with lipid droplets. CDC42, a facilitator of the G0/G1 to S phase transition (28), had increased protein but reduced transcript levels in colonic tumors; its reduced transcript levels predicted poorer survival (Fig. 3A–C). Similarly, TIP30, involved in DNA damage repair (29), had elevated protein levels but reduced transcript levels in colonic tumors; its reduced transcript levels predicted improved survival outcomes (Fig. 3A–C). Moreover, SFN, a regulator of G2/M checkpoint (30), had increased protein levels despite reduced transcript levels, although unrelated to patient survival (Fig. 3A–C). These findings implicated an important role for lipid droplet-associated regulators of the cell cycle in colon cancer growth.

**Fig. 3:**
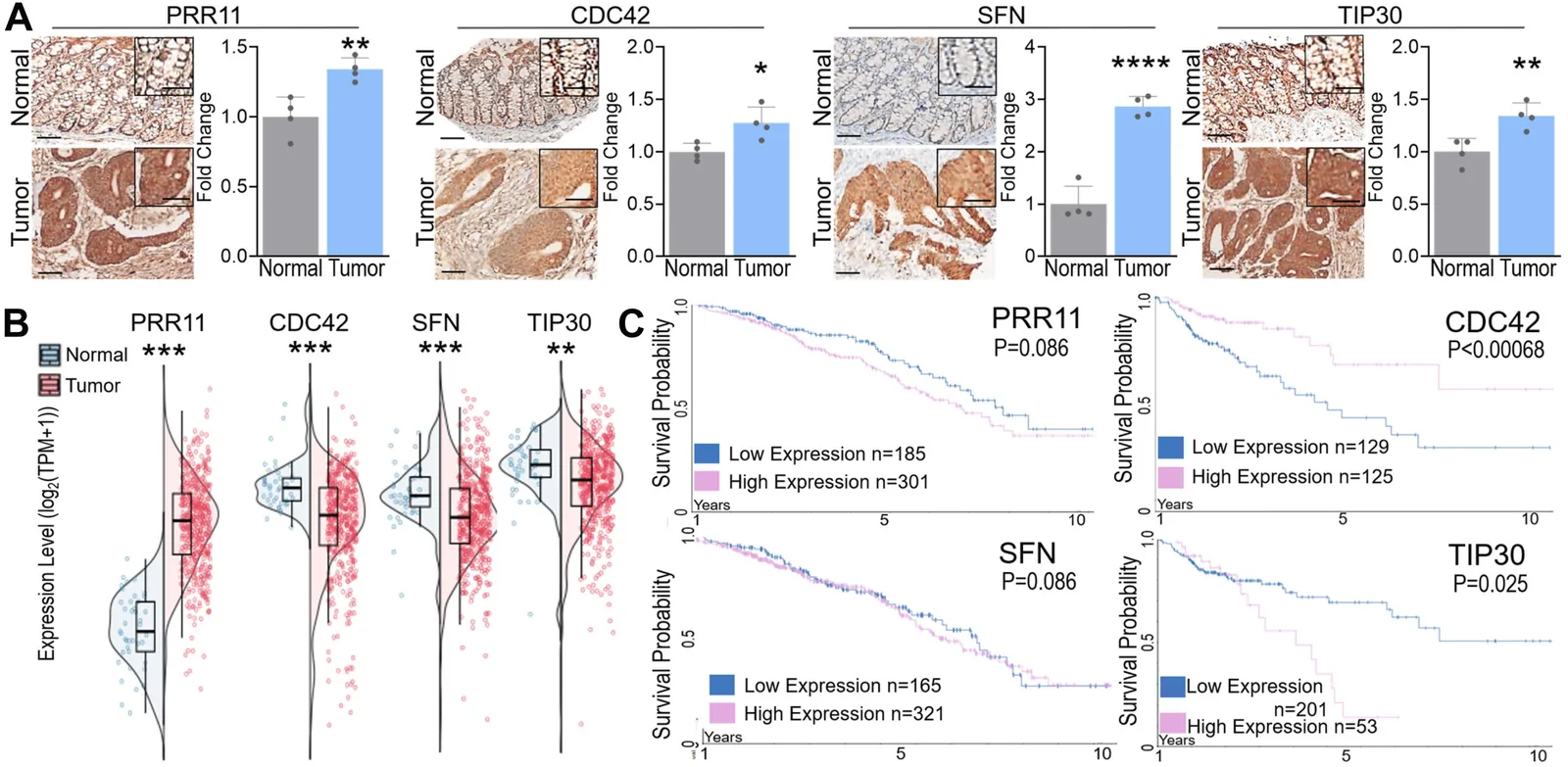
A: Lipid droplet-associated proteins regulating cell cycle, PRR11, CDC42, SFN, and TIP30, in human colonic tumors. Immunohistostaining of Stage 2 human colon cancer tumor tissue and adjacent normal (the scale bar represents 50 μm; inset 20 μm). Graphs represent densitometric analysis using ImageJ. Data are presented as mean ± SD (n = 4 patients, n = 4 average images per patient). Statistical significance was determined by unpaired *t* test; *P* < 0.05 was considered statistically significant (∗*P* < 0.05, ∗∗*P* < 0.01, ∗∗∗∗*P* < 0.0001 versus normal). B: Violin plots of *PRR11*, *CDC42*, *SFN*, and *TIP30* transcripts in colonic tumors versus normal samples in publicly available transcriptomes of colon cancer patients (TCGA via TIMER3, n = 478 tumor, n = 41 normal, ∗∗*P* < 0.01, ∗∗∗*P* < 0.001 versus normal). C: Kaplan-Meier survival probability of *PRR11*, *CDC42*, *SFN*, and *TIP30* in colon cancer transcriptomes (Human Protein Atlas validated TCGA dataset, *PRR11* n = 185 low expression and n = 301 high expression, total n = 486; *CDC42* n = 129 low expression and n = 125 high expression, total n = 254; *SFN* n = 165 low expression and n = 321 high expression, total n = 486; *TIP30* n = 201 low expression and n = 53 high expression, total n = 254). CDC42, Cell Division Cycle 42; PRR11, Proline Rich 11; SFN, Stratifin; TIP30, TAT-interacting Protein 30; TCGA, The Cancer Genome Atlas.

### Lipid droplet-associated cytoskeletal regulators are altered in human colon cancer

Several regulators of cytoskeletal dynamics, including Desmoplakin (DSP), MICAL2, LASP1 and CFL1, were identified to be associated with lipid droplets. DSP, an anchor for intermediate filaments that maintains cell-cell junctions (31), MICAL2, a regulator of actin dynamics during cell migration (32), LASP1, which promotes actin polymerization during cell migration 33, 34, and CFL1, responsible for actin filament turnover (35), were increased in protein levels in human colonic tumor tissue (Fig. 4A–D). In contrast, their transcript levels remained unchanged or decreased in colonic tumors and were not associated with patient survival (data not shown). Given their diverse roles in cytoskeletal organization, cell adhesion, and migration, these findings suggest that lipid droplet-associated cytoskeletal regulators may utilize lipid droplets to promote colon cancer growth, mechanisms of which need further investigation.

**Fig. 4:**
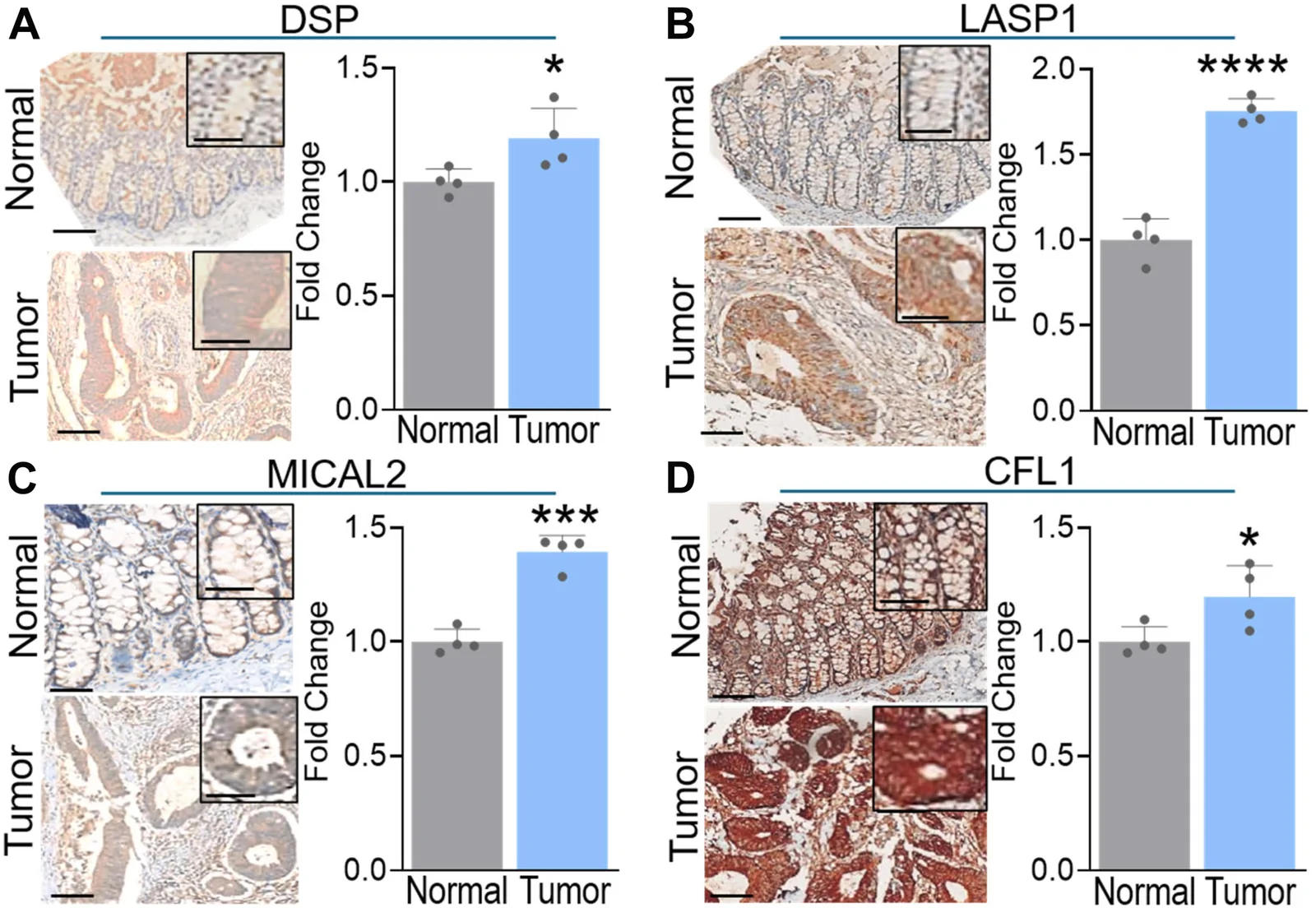
A−D: Lipid droplet-associated proteins regulating cytoskeleton dynamics, DSP, LASP1, MICAL2, and CFL1, in human colonic tumors. Immunohistostaining of Stage 2 human colon cancer tumor tissue and adjacent normal tissue (the scale bar represents 50 μm; inset 20 μm). Graphs represent densitometric analysis using ImageJ. Data are presented as mean ± SD (n = 4 patients, n = 4 average images per patient). Statistical significance was determined by unpaired *t* test; *P* < 0.05 was considered statistically significant (∗*P* < 0.05, ∗∗∗*P* < 0.001, ∗∗∗∗*P* < 0.0001 versus normal). CFL1, Cofilin 1; LASP1, LIM and SH3 protein 1; MICAL2, Molecule Interacting with CasL 2.

### Expression of lipid droplet-associated proteins in human colon cancer cells is facilitated by lipid droplet accumulation

We further examined the dependency of lipid droplet-associated protein expression on lipid droplets. Colon cancer cells, HCT116 and HT29, which have high capacity for lipid droplet accumulation 36, 37, were treated with a long-chain fatty acid, oleic acid, to induce lipid droplet accumulation (supplemental Fig. S2). In these treated cells, we demonstrated colocalization of ACSL3, ACSL4, PNPLA3, HILPDA, PRR11, CDC42, SFN, TIP30, DSP, LASP1, MICAL2, and CFL1 with lipid droplets (supplemental Fig. S3A–F). In addition, in HCT116 and HT29 cells, oleic acid treatment significantly increased the RNA and protein levels of lipid metabolism regulators, ACSL3, ACSL4, HILPDA, and PNPLA3, apart from ACSL3, whose RNA levels, while increased in HCT116 cells, were decreased in HT29 cells (Fig. 5A–D). We further demonstrated that oleic acid treatment significantly increased the protein levels of the cell cycle regulators PRR11, CDC42, SFN, and TIP30 in both HCT116 and HT29 cells, as well as the RNA levels of *PRR11*, whereas RNA levels of *CDC42*, *SFN*, and *TIP30* were increased only in HT29 cells (Fig. 6A–D). These cell-line specific responses to oleic acid treatment could be due to the distinct molecular and genetic characteristics of the HT29 and HCT116 cell lines (38). Furthermore, while RNA levels remained unchanged (data not shown), protein levels of cytoskeletal regulators DSP, LASP1, MICAL2, and CFL1 were increased in both HCT116 and HT29 cells in response to oleic acid treatment (Fig. 7A, B). These findings demonstrated that lipid droplet accumulation increased the expression of lipid droplet associated regulators of lipid metabolism, cell cycle progression, and cytoskeletal dynamics, supporting the role of lipid droplets in these cellular functions and in colon cancer cell growth.

**Fig. 5:**
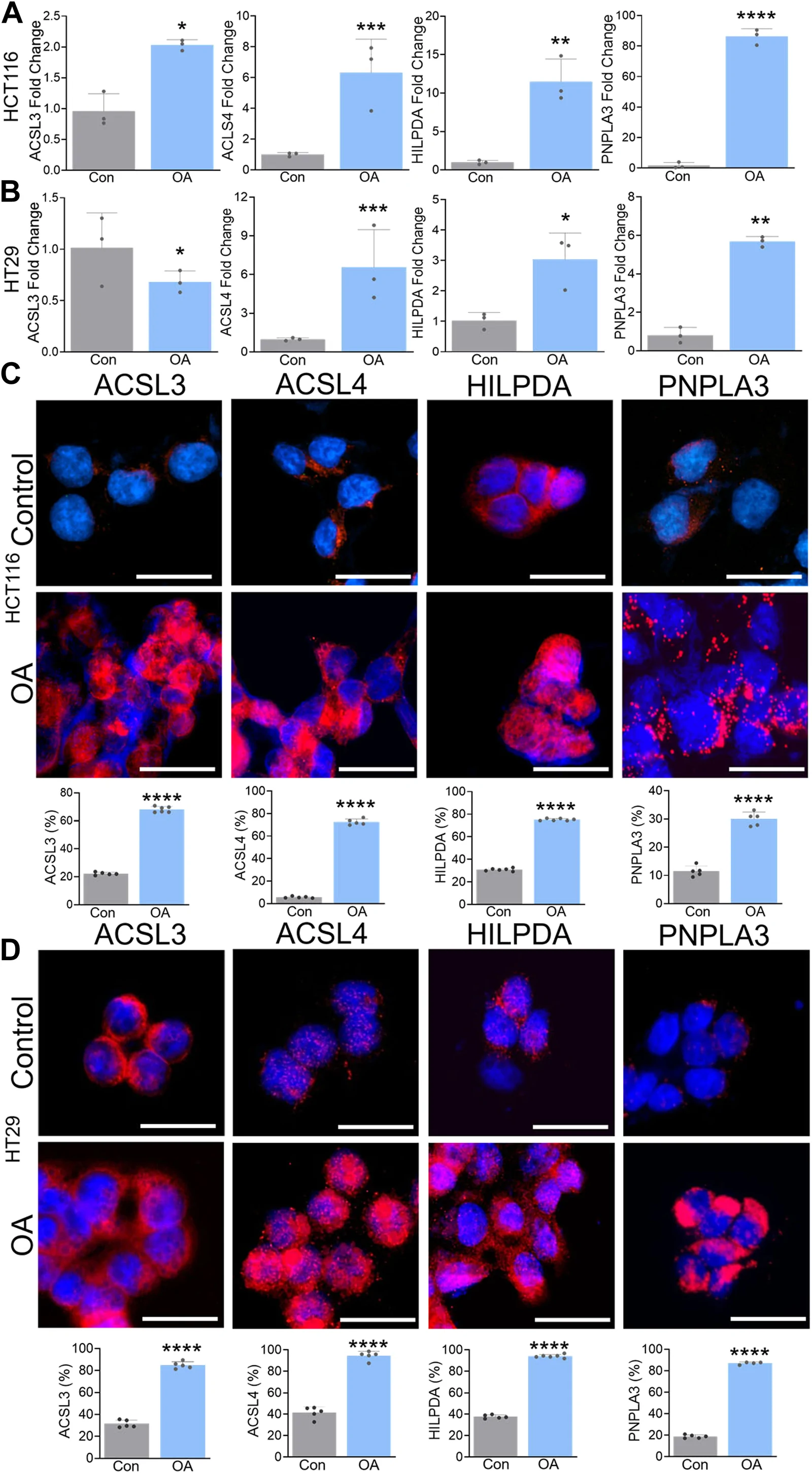
A, B: ACSL3, ACSL4, HILPDA, and PNPLA3 expression in human colon cancer cells stimulated by lipid droplet accumulation. RNA levels of *ACSL3*, *ACSL4*, *HILPDA*, and *PNPLA3* in human colon cancer cells, HCT116 and HT29, treated with oleic acid (OA, 300 μM) for 24 h (qPCR, n = 3, mean ± SD, ∗*P* < 0.05, ∗∗*P* < 0.01, ∗∗∗*P* < 0.001, ∗∗∗∗*P* < 0.0001 versus untreated control). Relative mRNA expression was calculated using the 2^−ΔΔCt^ method and normalized to the housekeeping *B2M* gene. C, D: ACSL3, ACSL4, HILPDA, and PNPLA3 protein levels in human colon cancer cells stimulated by lipid droplet accumulation. Immunofluorescence of ACSL3, ACSL4, HILPDA, and PNPLA3 protein in human colon cancer cells, HCT116 and HT29, treated with oleic acid (OA, 300 μM) for 24 h (the scale bar represents 15 μm). Graphs represent protein levels quantified as protein positive area (red) normalized to DAPI positive area (blue). Data are presented as mean ± SD (n = 4 wells per treatment, 5-6 images per group). Statistical significance was determined by unpaired *t* test; *P* < 0.05 was considered statistically significant (∗∗∗∗*P* < 0.0001 versus untreated control). ACSL, acyl-CoA synthetase; DAPI, 4',6-diamidino-2-phenylindole; HILPDA, hypoxia-inducible lipid droplet-associated protein; PNPLA3, phospholipase domain-containing protein 3; qPCR, quantitative polymerase chain reaction.

**Fig. 6:**
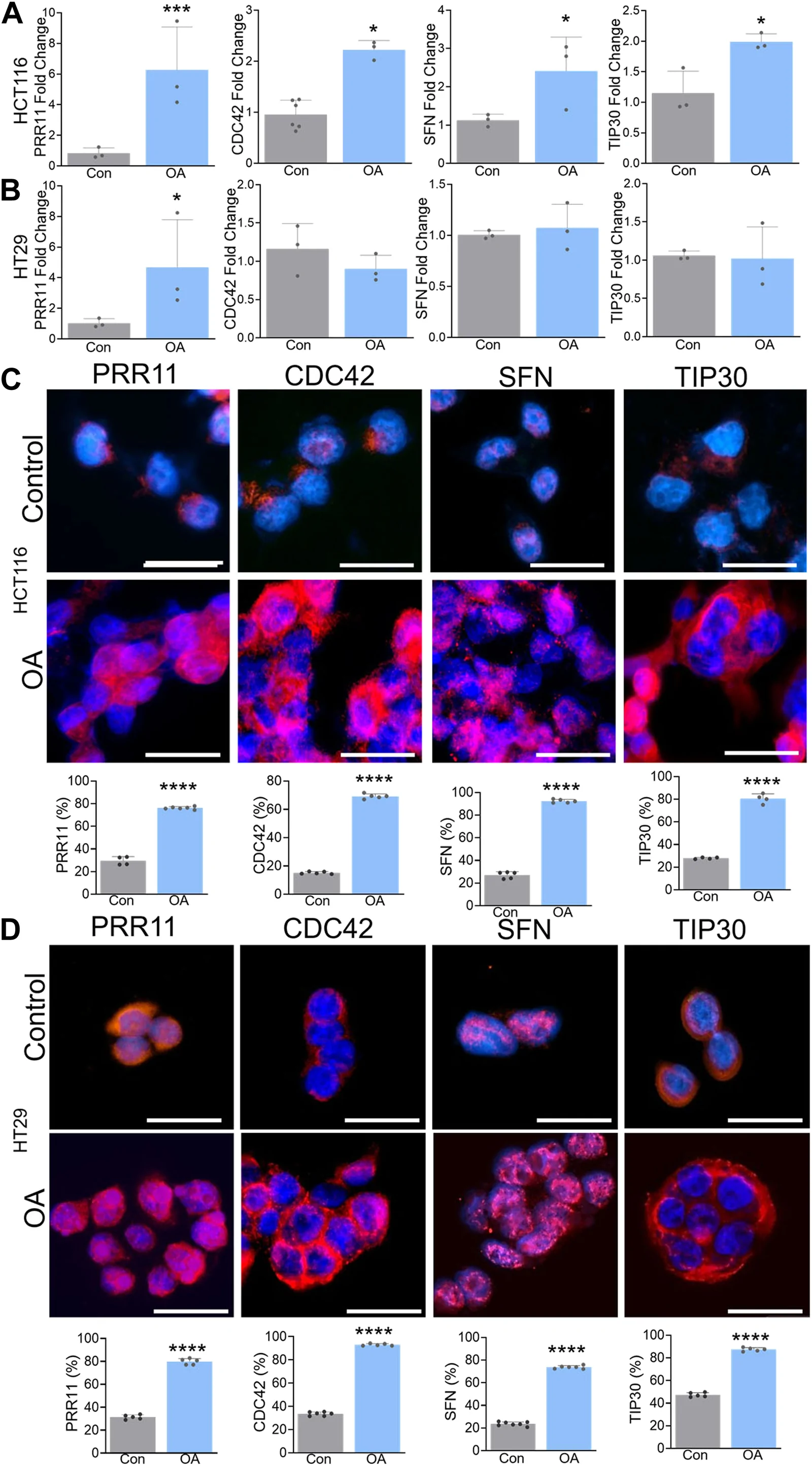
A, B: PRR11, CDC42, SFN, and TIP30 expression in human colon cancer cells stimulated by lipid droplet accumulation. RNA levels of *PRR11*, *CDC42*, *SFN*, and *TIP30* in human colon cancer cells, HCT116 and HT29, treated with oleic acid (OA, 300 μM) for 24 h (qPCR, n = 3, mean ± SD, ∗*P* < 0.05, ∗∗∗*P* < 0.001 versus control). Relative mRNA expression was calculated using the 2^−ΔΔCt^ method and normalized to the housekeeping gene (*B2M*). C, D: PRR11, CDC42, SFN, and TIP30 protein levels in human colon cancer cells stimulated by lipid droplet accumulation. Immunofluorescence of PRR11, CDC42, SFN, and TIP30 protein in human colon cancer cells, HCT116 and HT29, treated with oleic acid (OA, 300 μM) for 24 h (the scale bar represents 15 μm). Graphs represent protein levels quantified as protein positive area (red) normalized to DAPI positive area (blue). Data are presented as mean ± SD (n = 4 wells per treatment, 5-6 images per group). Statistical significance was determined by unpaired *t* test; *P* < 0.05 was considered statistically significant (∗∗∗∗*P* < 0.0001 versus untreated control). CDC42, Cell Division Cycle 42; DAPI, 4',6-diamidino-2-phenylindole; PRR11, Proline Rich 11; qPCR, quantitative polymerase chain reaction; SFN, Stratifin; TIP30, TAT-interacting Protein 30.

**Fig. 7:**
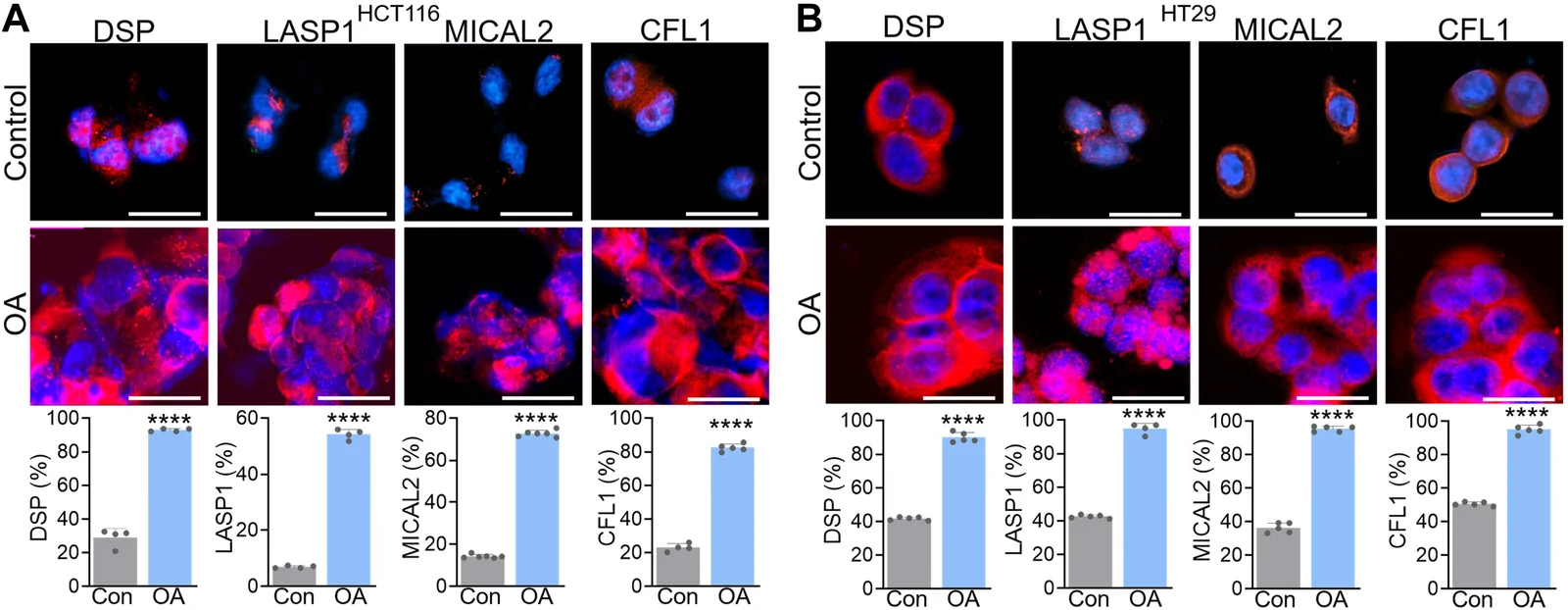
A, B: DSP, LASP1, MICAL2 and CFL1 protein levels in human colon cancer cells stimulated by lipid droplet accumulation. Immunofluorescence of DSP, LASP1, MICAL2 and CFL1 in human colon cancer cells, HCT116 and HT29, treated with oleic acid (OA, 300 μM) for 24 h (the scale bar represents 15 μm). Graphs represent protein levels quantified as protein-positive area (red) normalized to DAPI-positive area (blue). Data are presented as mean ± SD (n = 4 wells per treatment, 4-6 images per group). Statistical significance was determined by unpaired *t* test; *P* < 0.05 was considered statistically significant (∗∗∗∗*P* < 0.0001 versus untreated control). CFL1, Cofilin 1; DAPI, 4',6-diamidino-2-phenylindole; LASP1, LIM and SH3 protein 1; MICAL2, Molecule Interacting with CasL 2.

### Role of lipid droplet-associated proteins in colon cancer cell growth and migration

To evaluate the functional role of lipid droplet-associated proteins in cancer cell growth and migration, we selectively knocked down representative genes regulating lipid metabolism, ACSL4, cell cycle progression, PRR11, and cytoskeletal dynamics, LASP1 and MICAL2, in colon cancer cells. Efficiency of siRNA knockdown was approximately 50% in both HCT116 and HT29 cells relative to scrambled siRNA (supplemental Fig. S4). The knockdown of ACSL4, PRR11, LASP1, or MICAL2 significantly reduced oleic acid induced lipid droplet accumulation in both cell lines (supplemental Fig. S5), indicating that these proteins also contribute to lipid droplet accumulation. Further functional studies demonstrated that knockdown of each of these proteins significantly attenuated oleic acid stimulated cell proliferation in both HCT116 and HT29 cells, which was not seen in control (untreated cells) (Fig. 8A, B), supporting a lipid droplet dependent role of these proteins in colon cancer growth. Cell cycle assessment further demonstrated the impact of these proteins on the growth of HCT116 cells. Knockdown of ACSL4 resulted in G0/G1 arrest, whereas knockdown of PRR11 and LASP1 resulted in G0/G1 and S-phase arrest, respectively. MICAL2 knockdown disrupted S-phase progression (Fig. 8C). Moreover, knockdown of these proteins significantly reduced the migration capacity of HCT116 cells under oleic acid treatment (Fig. 8D).

**Fig. 8:**
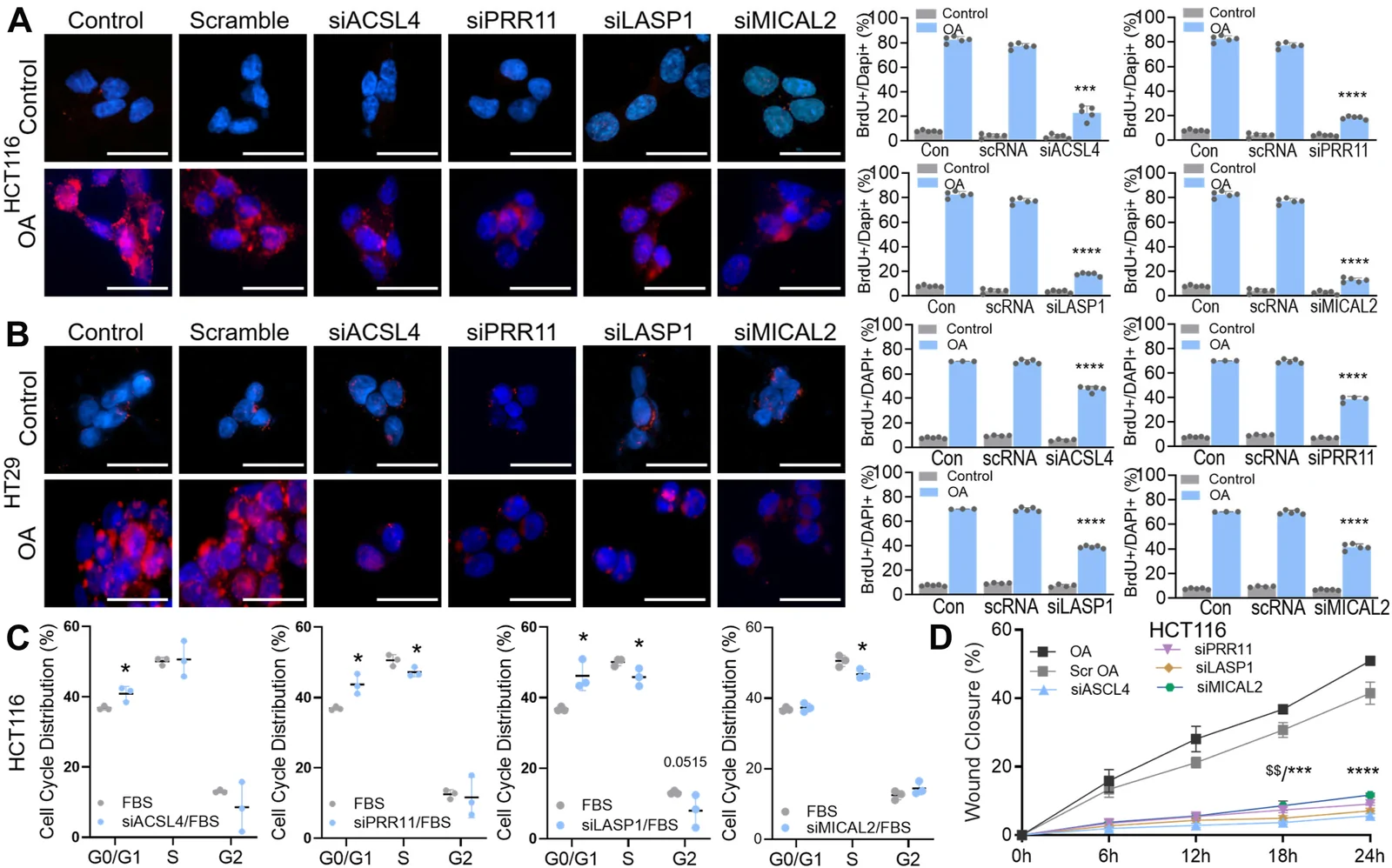
A, B: Proliferation of human colon cancer cells with knockdown of ACSL4, PRR11, LASP1, or MICAL2. Immunofluorescence staining for BrdU in human colon cancer cells, HCT116 and HT29, transfected with siRNA targeting ACSL4, PRR11, LASP1, or MICAL2 (scrambled siRNA as a control) and treated with oleic acid (OA, 300 μM) for 6 h, followed by BrdU labeling (10 μM) during the final 3 h (the scale bar represents 15 μm). Graphs represent BrdU-positive area normalized to DAPI-positive area. Data are presented as mean ± SD (n = 4 wells per treatment, 5-6 images per group). Statistical significance was determined by two-way ANOVA with Tukey’s multiple comparisons; *P* < 0.05 was considered statistically significant (∗∗∗*P* < 0.001, ∗∗∗∗*P* < 0.0001 compared to OA-treated scrambled siRNA control). C: Cell cycle of human colon cancer cells with knockdown of ACSL4, PRR11, LASP1, or MICAL2. Cell cycle analysis in human colon cancer cells, HCT116, following knockdown of ACSL4, PRR11, LASP1, or MICAL2 by siRNA (scrambled siRNA as a control). Graphs represent the percentage of cells in each phase of the cell cycle, and data are presented as mean ± SD (n = 3 per siRNA). Statistical significance was determined by unpaired *t* test; *P* < 0.05 was considered statistically significant (∗*P* < 0.05 versus control (FBS treatment)). D: Migration of human colon cancer cells with knockdown of ACSL4, PRR11, LASP1, or MICAL2. Migration assay of human colon cancer cells, HCT116, following knockdown of ACSL4, PRR11, LASP1, or MICAL2. A uniform scratch was generated in confluent monolayers, followed by treatment with oleic acid (OA, 300 μM). Wound closure was monitored for 24 h with images acquired at 6 h intervals (BioSpa–Cytation5). Data are presented as mean ± SD. Statistical significance was determined by two-way ANOVA with Tukey’s multiple comparisons; *P* < 0.05 was considered statistically significant (n = 3 wells per treatment, mean ± SD, ∗∗∗*P* < 0.001, ∗∗∗∗*P* < 0.0001 siRNA versus OA-treated scrambled siRNA control, $$*P* < 0.01 siACSL4 versus OA-treated scrambled siRNA control). ACSL, acyl-CoA synthetase; DAPI, 4',6-diamidino-2-phenylindole; LASP1, LIM and SH3 protein 1; MICAL2, Molecule Interacting with CasL 2; PRR11, Proline Rich 11.

Next, we examined whether the expression of *ACSL4* and *PRR11* changes with tumor progression (Stages 1–4) and CMS: CMS1-microsatellite instability, CMS2- canonical epithelial, CMS3- metabolic, and CMS4- mesenchymal subtypes in a publicly available colon cancer transcriptomic dataset. *ACSL4* and *PRR11* showed increased transcript levels with advancing tumor stage. In addition, they have distinct expression patterns across the colon cancer Consensus Molecular Subtypes, showing that *ACSL4* and *PRR11* were highly expressed in the canonical epithelial and mesenchymal subtypes than in the microsatellite instability-associated subtype (Fig. 9A, B). Together, these findings demonstrated that lipid droplet-associated proteins promote colon cancer cell proliferation, cell cycle progression, and migration, suggesting disease stage and subtype-specific contributions to colon cancer growth.

**Fig. 9:**
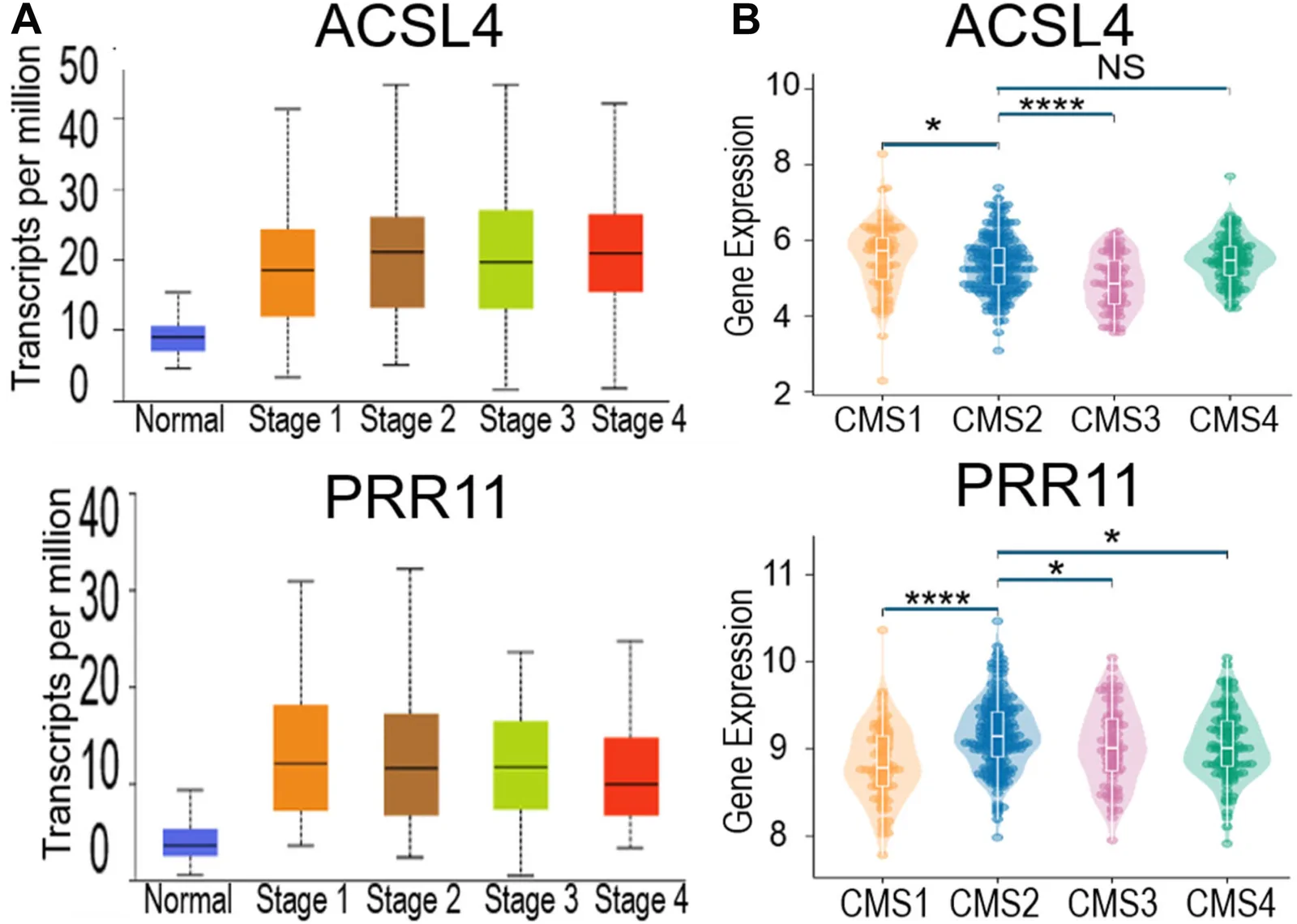
A: *ACSL4* and *PRR11* transcript levels with colon cancer stage progression. *ACSL4* and *PRR11* transcript levels across colon cancer Stages 1–4 versus normal colon in a publicly available colon cancer transcriptomic dataset (TCGA, Normal n = 41, Stage 1 n = 45, Stage 2 n = 110, Stage 3 n = 80, Stage 4 n = 39). B: *ACSL4* and *PRR11* transcript levels across colon cancer subtypes. Violin plots showing ACSL4 and PRR11 transcript expression across the Consensus Molecular Subtypes (CMS) of colon cancer: CMS1 (immune-microsatellite instability), CMS2 (canonical), CMS3 (metabolic), and CMS4 (mesenchymal). Statistical significance was determined by unpaired *t* test; *P* < 0.05 was considered statistically significant (TCGA, CMS1 n = 90, CMS2 n = 56, CMS3 n = 83, CMS4 n = 50, mean ± SD, ∗*P* < 0.05, ∗∗∗∗*P* < 0.0001; NS, not significant versus CMS2 control). ACSL, acyl-CoA synthetase; PRR11, Proline Rich 11; TCGA, The Cancer Genome Atlas.

## Discussion

The remodeling of lipid metabolism during colon cancer growth involves dysregulation of oncogenic pathways and organelles, including lipid droplets 5, 39, 40, 41. Our findings support lipid droplets as a central orchestrator of colon cancer progression, integrating lipid metabolism, cell cycle regulation, and cytoskeletal remodeling to support tumor growth. We generated a transcriptional signature based on a published publicly available lipid droplet proteome (19) representing 151 proteins associated with lipid droplets, which was significantly enriched in human colon cancer, distinguishing tumor from normal tissue. Several of these lipid droplet-associated proteins involved in lipid metabolism, cell cycle, and cytoskeletal organization were increased in colonic tumors, with some of them correlating with poorer patient survival. Furthermore, induced lipid droplet accumulation in colon cancer cells elevated the expression of these proteins. Knockdown of representative regulators of lipid metabolism, cell cycle, and cytoskeletal dynamics reduced proliferation, cell cycle progression, and migration, further emphasizing their significance in tumorigenesis. The dysregulation of lipid droplet-associated proteins in colonic tumors underscores their potential clinical utility as biomarkers and therapeutic targets in colon cancer patients.

The lipid metabolism regulators, ACSL3 and ACSL4, are increased in several cancers and promote tumor progression through multiple mechanisms including metabolic remodeling, ferroptosis, and angiogenesis 42, 43, 44, 45, 46, 47. In lung and colon cancer, ACSL3 promotes tumor growth through oncogenic KRAS or TGFβ1 dependent epithelial-mesenchymal transition 48, 49. Our findings showed differential ACSL3 expression among colon cancer cell lines, which suggests that its expression may depend on p53 mutations as well as metabolic and ferroptosis pathways (38). Moreover, increased HILPDA promotes tumor survival under hypoxia, partly by inhibiting ATGL, which enhances lipid retention in lipid droplets, further sustaining cancer cell survival 50, 51. Consistent with this role, increased HILPDA expression has also been linked to renal cell carcinoma through activation of Wnt signaling (50). Similarly, PNPLA3 overexpression or mutations have been reported as a critical driver of liver cancer 52, 53. Although the upstream mechanisms regulating these lipid droplet-associated proteins remain to be defined, their increased expression may involve lipid-responsive transcription factors, including PPARs and sterol regulatory element-binding proteins (SREBPs) (54). Furthermore, while lipid droplet initiation and growth involve the endoplasmic reticulum, Golgi apparatus, and cytosol 8, 9, the subcellular compartments in which these proteins colocalize with lipid droplets remain unexamined. We previously demonstrated that, in TNF stimulated colon cancer cells, lipid droplets colocalize with IL-8 within the endoplasmic reticulum, Golgi apparatus, and cytosolic compartments (55), suggesting a potential role for lipid droplets in coordinating protein trafficking, processing, or maturation. Collectively, these findings support a model in which lipid droplets function as dynamic signaling and regulatory hubs that coordinate interactions among organelles, regulators of lipid metabolism, and signaling pathways during tumorigenesis.

Limited findings suggest a link between lipid droplet dynamics and cell cycle 56, 57, though the underlying mechanisms in cancer growth remain poorly understood. We demonstrated a mechanistic link between lipid droplets and cell cycle progression in colon cancer cells, mediated via loss of FOXO3 dependent p27Kip1 regulation 11, 12, 13. It has been indicated that cell cycle regulators associated with lipid droplets may act as metabolic sensors (58), enabling cancer cells to monitor the availability of the fuel required for their rapid growth. Lipid droplet-associated cell cycle regulator PRR11 is upregulated in multiple cancers, promoting proliferation, migration, and cell cycle progression, in part, through the ERK/AKT signaling (59). Beyond its role in cell cycle regulation, CDC42 also regulates the cytoskeletal dynamics that facilitate cancer cell migration 28, 60. SFN also has multiple functions in promoting cancer growth, such as gene methylation, cytoskeletal remodeling, and apoptosis 61, 62, 63. Our findings showed dysregulated TIP30 expression in colon cancer, highlighting its complex regulation and roles in colonic tumors. Together, these findings support a mechanistic link between lipid droplets and cell cycle in exacerbating colonic tumorigenesis. Additional processes, including impaired DNA damage responses associated with lipid droplet accumulation, may also contribute to this phenotype (64). Further studies are needed to define how cell cycle regulators interact with lipid droplets and determine whether these interactions represent therapeutic opportunities for colon cancer treatments.

We also identified several regulators of cytoskeletal dynamics that are associated with lipid droplets, including DSP, MICAL2, LASP1, and CFL1, which were dysregulated in colonic tumors. Emerging findings reveal that remodeling of cytoskeletal proteins, critical for cancer invasion and metastasis, may depend on their association with lipid droplets 65, 66. Conversely, limited studies in triple-negative breast cancer suggest that lipid storage may suppress metastasis under selected conditions by restricting fatty acid availability for mitochondrial oxidation 13, 66, indicating that lipid droplet dependent metastatic progression may vary according to tumor type, metabolic state, and disease stage. Furthermore, DSP has been implicated in colon cancer growth, although its mechanistic contribution remains incompletely defined (67). We found increased DSP protein levels in colonic tumors despite unchanged transcript levels, raising the possibility that lipid droplets may sequester and stabilize DSP protein rather than simply increasing its expression. MICAL2 has been implicated in cancer and linked to KRAS signaling and epithelial-mesenchymal transition (32). This suggests that MICAL2 mediated actin remodeling could influence lipid droplet mobility, organelle interactions, and intracellular distribution, although the role of MICAL2 in lipid droplet dynamics requires further investigation. Furthermore, increased MICAL2 promotes CDC42 mediated E-cadherin degradation, enhancing gastric cancer cell migration (68). This localization of both MICAL2 and CDC42 to lipid droplets is particularly intriguing, as it supports that lipid droplets may integrate pathways controlling cytoskeletal remodeling and cell cycle progression. In addition, increased LASP1 has been reported in primary tumors, where it facilitates migration and inflammation (34). In colon cancer, LASP1 increases Nanog expression via YAP1 activation, facilitating cancer cell growth (69). The identification of LASP1 as a lipid droplet-associated protein further supports an interaction between lipid droplets and regulators of cytoskeletal remodeling during tumorigenesis. Similarly, CFL1, which is overexpressed in several cancers and promotes epithelial-to-mesenchymal transition 35, 70, was found to be associated with lipid droplets. These findings suggest that regulators of cytoskeletal remodeling and cell cycle progression may utilize lipid droplets to coordinate their functions during cancer growth. Moreover, recruitment or sequestration of cytoskeletal regulators at lipid droplets could alter their availability for actin remodeling, adhesion turnover, and directional migration, the mechanism of which remains to be directly established. Thus, the functional consequences of lipid droplet-associated cytoskeletal proteins likely depend on the specific protein, tumor type, metabolic state, and stage of disease.

An important finding of this study is the increased protein abundance of several lipid droplet-associated proteins in the absence of corresponding changes in transcript expression. This discordance was seen for *CDC42*, *SFN*, *TIP30*, *DSP*, *LASP1*, *MICAL2*, and *CFL1* in human colon cancer, suggesting that protein levels may be regulated through posttranscriptional or posttranslational mechanisms rather than transcription alone. Moreover, our functional studies demonstrated that lipid droplet-associated proteins actively support colon cancer cell growth. Furthermore, the progressive increase in *ACSL4* and *PRR11* expression with advancing tumor stage, together with their distinct expression patterns across CMS, further supports their clinical relevance in colon cancer progression. The diverse expression patterns of these lipid droplet-associated proteins among biologically distinct CMS subtypes suggest that lipid droplet dependent mechanisms are differentially engaged in colon tumorigenesis. These findings suggest that lipid droplet-associated proteins may contribute to the molecular heterogeneity of colon cancer, requiring further mechanistic investigation.

It is important to consider several limitations of this study, such as we only assessed lipid droplet-associated proteins without direct lipidomic measurement, including triglycerides and their turnover, which reflect metabolic adaptation of tumor cells 3, 4. In addition, whether these lipid droplet-associated proteins are recruited directly to lipid droplets or remain in proximity will require biochemical validation. Despite this uncertainty, our findings support a model in which lipid droplets function as dynamic metabolic hubs that coordinate lipid availability with regulators of cell cycle progression and cytoskeletal remodeling. Furthermore, functional validation was limited to four representative proteins, although additional members of the LDs_151_ signature may also contribute to colon tumorigenesis by different mechanisms. Finally, although oleic acid treated colon cancer cells provide a relevant model for studying lipid droplet dynamics and activate similar molecular pathways seen during tumorigenesis 12, 13, they do not fully recapitulate the complexity of the tumors. Therefore, validation in genetically engineered mouse models and patient derived organoids will be important to establish the physiological significance of these findings.

## Conclusion

Lipid droplets are dynamic organelles that play a central role in metabolic remodeling during cancer growth 6, 7. These findings support the emerging concept that lipid droplets function as regulatory hubs integrating lipid metabolism, cell cycle progression, and cytoskeletal remodeling to promote colon cancer growth. Collectively, these findings expand our understanding of lipid droplet biology in colonic tumorigenesis and identify lipid droplet-associated proteins as promising biomarkers and potential therapeutic targets for colon cancer patients.

## Data availability

We will make our published data, analytic methods, and study materials available to other researchers.

## Supplemental data

This article contains supplemental data.

## Conflict of interest

The authors declare that they have no conflicts of interest with the contents of this article.

## Supplemental data

Supplemental Fig. 1 Lipid droplet-coat protein PLIN2 in human colonic tumors. Immunohistostaining of PLIN2 in Stage 2 human colon cancer tissue and matched adjacent normal tissue (the scale bar represents 50 μm; inset 20 μm). Graphs represent densitometric analysis using ImageJ. Data are presented as mean ± SD (n = 4 patients, n = 11–12 images per patient). Statistical significance was determined by unpaired *t* test; P < 0.05 was considered statistically significant (∗∗∗∗P < 0.0001 versus normal control). PLIN2, Perilipin 2.

Supplemental Fig. 2 Lipid droplet accumulation stimulated in human colon cancer cells. Immunofluorescence of lipid droplets in human colon cancer cells, HCT116 and HT29, treated with oleic acid (OA, 300 μM) for 24 h (the scale bar represents 15 μm). Graphs represent lipid droplet levels quantified as BODIPY-positive area (green) normalized to DAPI-positive area (blue); data are presented as mean ± SD (n = 4, 6-11 images per group). Statistical significance was determined by unpaired *t* test; P < 0.05 was considered statistically significant (∗∗∗P < 0.001, ∗∗∗∗P < 0.0001 versus untreated control). DAPI, 4',6-diamidino-2-phenylindole.

Supplemental Fig. 3 A–F: Colocalization of ACSL3, ACSL4, HILPDA, PNPLA3, PRR11, CDC42, SFN, TIP30, DSP, LASP1, MICAL2, or CFL1 with lipid droplets. Immunofluorescence of human colon cancer cells, HCT116 and HT29, treated with oleic acid (OA, 300 μM) for 24 h and stained for ACSL3, ACSL4, HILPDA, PNPLA3, PRR11, CDC42, SFN, TIP30, DSP, LASP1, MICAL2, or CFL1 (red), and for lipid droplets (BODIPY-green). Inserts show enlarged views of the indicated regions (the scale bar represents 15 μm in main image, 5 μm in insert). ACSL, acyl-CoA synthetase; HILPDA, hypoxia-inducible lipid droplet-associated protein; PNPLA3, phospholipase domain-containing protein 3; PRR11, Proline Rich 11; CDC42, Cell Division Cycle 42; SFN, Stratifin; TIP30, TAT-interacting Protein 30; LASP1, LIM and SH3 protein 1; MICAL2, Molecule Interacting with CasL 2; CFL1, Cofilin 1.

Supplemental Fig. 4 A–H: Knockdown of ACSL4, PRR11, LASP1, or MICAL2 in human colon cancer cells. Immunoblot of ACSL4, PRR11, LASP1, or MICAL2 protein levels in human colon cancer cells, HCT116 and HT29, following their knockdown with siRNA or scrambled (siRNA) control. Actin is a loading control. Graphs represent densitometry of ACSL4, PRR11, LASP1, or MICAL2. Statistical significance was determined by one-way ANOVA with Tukey’s multiple comparisons; P < 0.05 was considered statistically significant (n = 3, mean ± SD, ∗∗∗P < 0.001, ∗∗∗∗P < 0.0001 versus scrambled siRNA control). ACSL, acyl-CoA synthetase; PRR11, Proline Rich 11; LASP1, LIM and SH3 protein 1; MICAL2, Molecule Interacting with CasL 2.

Supplemental Fig. 5 A, B: Stimulated lipid droplet accumulation in human colon cancer cells with knockdown of ACSL4, PRR11, LASP1, or MICAL2. Immunofluorescence of human colon cancer cells, HCT116 and HT29, transfected with siRNA targeting ACSL4, PRR11, LASP1, or MICAL2 (scrambled siRNA control), treated with oleic acid (OA, 300 μM) for 24 h and stained for lipid droplets (BODIPY). Quantification of lipid droplet accumulation was measured as BODIPY-positive area (green) normalized to DAPI-positive area (blue); data are presented as mean ± SD (n = 4 per group, 5-6 images per group). Statistical significance was determined by two-way ANOVA followed by Tukey’s multiple comparisons. P < 0.05 was considered statistically significant (∗∗P < 0.01, ∗∗∗P < 0.001, ∗∗∗∗P < 0.0001 versus OA-treated scrambled siRNA control). ACSL, acyl-CoA synthetase; PRR11, Proline Rich 11; LASP1, LIM and SH3 protein 1; MICAL2, Molecule Interacting with CasL 2; DAPI, 4',6-diamidino-2-phenylindole.

## References

[1] Raskov H., Soby J.H., Troelsen J., Bojesen R.D., Gogenur I. (2020). Driver gene mutations and epigenetics in colorectal cancer. Ann. Surg..

[2] Bray F., Ferlay J., Soerjomataram I., Siegel R.L., Torre L.A., Jemal A. (2018). Global cancer statistics 2018: GLOBOCAN estimates of incidence and mortality worldwide for 36 cancers in 185 countries. CA Cancer J. Clin..

[3] Pakiet A., Kobiela J., Stepnowski P., Sledzinski T., Mika A. (2019). Changes in lipids composition and metabolism in colorectal cancer: a review. Lipids Health Dis..

[4] Salita T., Rustam Y.H., Mouradov D., Sieber O.M., Reid G.E. (2022). Reprogrammed lipid metabolism and the lipid-associated hallmarks of colorectal cancer. Cancers (Basel).

[5] Petan T. (2020). Lipid droplets in cancer. Rev. Physiol. Biochem. Pharmacol..

[6] Dalhaimer P. (2019). Lipid droplets in disease. Cells.

[7] Jin Y., Tan Y., Wu J., Ren Z. (2023). Lipid droplets: a cellular organelle vital in cancer cells. Cell Death Discov..

[8] Welte M.A. (2015). Expanding roles for lipid droplets. Curr. Biol..

[9] Olzmann J.A., Carvalho P. (2019). Dynamics and functions of lipid droplets. Nat. Rev. Mol. Cell Biol..

[10] Straub B.K., Herpel E., Singer S., Zimbelmann R., Breuhahn K., Macher-Goeppinger S. (2010). Lipid droplet-associated PAT-proteins show frequent and differential expression in neoplastic steatogenesis. Mod. Pathol..

[11] Qi W., Fitchev P.S., Cornwell M.L., Greenberg J., Cabe M., Weber C.R. (2013). FOXO3 growth inhibition of colonic cells is dependent on intraepithelial lipid droplet density. J. Biol. Chem..

[12] Iftikhar R., Penrose H.M., King A.N., Samudre J.S., Collins M.E., Hartono A.B. (2021). Elevated ATGL in colon cancer cells and cancer stem cells promotes metabolic and tumorigenic reprogramming reinforced by obesity. Oncogenesis.

[13] Ghimire J., Collins M.E., Snarski P., King A.N., Ruiz E., Iftikhar R. (2025). Obesity-facilitated Colon cancer progression is mediated by increased diacylglycerol O-Acyltransferases 1 and 2 levels. Gastroenterology.

[14] Chapman K.D., Aziz M., Dyer J.M., Mullen R.T. (2019). Mechanisms of lipid droplet biogenesis. Biochem. J..

[15] Kilwein M.D., Welte M.A. (2019). Lipid droplet motility and organelle contacts. Contact (Thousand Oaks).

[16] Cases S., Smith S.J., Zheng Y.W., Myers H.M., Lear S.R., Sande E. (1998). Identification of a gene encoding an acyl CoA:diacylglycerol acyltransferase, a key enzyme in triacylglycerol synthesis. Proc. Natl. Acad. Sci. U. S. A..

[17] Vegliante R., Di Leo L., Ciccarone F., Ciriolo M.R. (2018). Hints on ATGL implications in cancer: beyond bioenergetic clues. Cell Death Dis..

[18] Zimmermann R., Strauss J.G., Haemmerle G., Schoiswohl G., Birner-Gruenberger R., Riederer M. (2004). Fat mobilization in adipose tissue is promoted by adipose triglyceride lipase. Science.

[19] Bersuker K., Peterson C.W.H., To M., Sahl S.J., Savikhin V., Grossman E.A. (2018). A proximity labeling strategy provides insights into the composition and dynamics of lipid droplet proteomes. Dev. Cell.

[20] Cui H., Zhao G., Lu Y., Zuo S., Duan D., Luo X. (2025). TIMER3: an enhanced resource for tumor immune analysis. Nucleic Acids Res..

[21] Uhlén M., Fagerberg L., Hallström B.M., Lindskog C., Oksvold P., Mardinoglu A. (2015). Proteomics. Tissue-based map of the human proteome. Science.

[22] Ghimire J., Iftikhar R., Penrose H.M., Snarski P., Ruiz E., Savkovic S.D. (2023). FOXO3 deficiency in neutrophils drives colonic inflammation and tumorigenesis. Int. J. Mol. Sci..

[23] Iftikhar R., Penrose H.M., King A.N., Kim Y., Ruiz E., Kandil E. (2022). FOXO3 expression in macrophages is lowered by a high-fat diet and regulates colonic inflammation and tumorigenesis. Metabolites.

[24] Consortium U. (2025). UniProt: the universal protein knowledgebase in 2025. Nucleic Acids Res..

[25] Gimm T., Wiese M., Teschemacher B., Deggerich A., Schodel J., Knaup K.X. (2010). Hypoxia-inducible protein 2 is a novel lipid droplet protein and a specific target gene of hypoxia-inducible factor-1. FASEB J..

[26] Chamoun Z., Vacca F., Parton R.G., Gruenberg J. (2013). PNPLA3/adiponutrin functions in lipid droplet formation. Biol. Cell.

[27] Ji Y., Xie M., Lan H., Zhang Y., Long Y., Weng H. (2013). PRR11 is a novel gene implicated in cell cycle progression and lung cancer. Int. J. Biochem. Cell Biol..

[28] Maruta H., Nheu T.V., He H., Hirokawa Y. (2003). Rho family-associated kinases PAK1 and rock. Prog. Cell Cycle Res..

[29] Fong S., King F., Shtivelman E. (2010). CC3/TIP30 affects DNA damage repair. BMC Cell Biol..

[30] Zhang B., Zhou B., Huang G., Huang J., Lin X., Li Z. (2024). Nitidine chloride inhibits G2/M phase by regulating the p53/14-3-3 Sigma/CDK1 axis for hepatocellular carcinoma treatment. Heliyon.

[31] Bornslaeger E.A., Corcoran C.M., Stappenbeck T.S., Green K.J. (1996). Breaking the connection: displacement of the desmosomal plaque protein desmoplakin from cell-cell interfaces disrupts anchorage of intermediate filament bundles and alters intercellular junction assembly. J. Cell Biol..

[32] Mariotti S., Barravecchia I., Vindigni C., Pucci A., Balsamo M., Libro R. (2016). MICAL2 is a novel human cancer gene controlling mesenchymal to epithelial transition involved in cancer growth and invasion. Oncotarget.

[33] Butt E., Gambaryan S., Gottfert N., Galler A., Marcus K., Meyer H.E. (2003). Actin binding of human LIM and SH3 protein is regulated by cGMP- and cAMP-dependent protein kinase phosphorylation on serine 146. J. Biol. Chem..

[34] Butt E., Howard C.M., Raman D. (2022). LASP1 in cellular signaling and gene expression: more than just a cytoskeletal regulator. Cells.

[35] Xu J., Huang Y., Zhao J., Wu L., Qi Q., Liu Y. (2021). Cofilin: a promising protein implicated in cancer metastasis and apoptosis. Front. Cell Dev. Biol..

[36] Penrose H., Heller S., Cable C., Makboul R., Chadalawada G., Chen Y. (2016). Epidermal growth factor receptor mediated proliferation depends on increased lipid droplet density regulated via a negative regulatory loop with FOXO3/Sirtuin6. Biochem. Biophys. Res. Commun..

[37] Cotte A.K., Aires V., Fredon M., Limagne E., Derangere V., Thibaudin M. (2018). Lysophosphatidylcholine acyltransferase 2-mediated lipid droplet production supports colorectal cancer chemoresistance. Nat. Commun..

[38] Loughran R.M., Emerling B.M. (2022). Mechanistic roles of mutant p53 governing lipid metabolism. Adv. Biol. Regul..

[39] Snaebjornsson M.T., Janaki-Raman S., Schulze A. (2020). Greasing the wheels of the cancer machine: the role of lipid metabolism in cancer. Cell Metab..

[40] Fernandez L.P., Gomez de Cedron M., Ramirez de Molina A. (2020). Alterations of lipid metabolism in cancer: implications in prognosis and treatment. Front. Oncol..

[41] Chakraborty S., Kumar A.S., Banerjee S. (2025). Lipids: driving forces in the underlying biology of carcinogenesis. ACS Pharmacol. Transl. Sci..

[42] Jiang Y., Zhang M., Sun M. (2025). ACSL4 at the helm of the lipid peroxidation ship: a deep-sea exploration towards ferroptosis. Front. Pharmacol..

[43] Feng S., Rao Z., Zhang J., She X., Chen Y., Wan K. (2023). Inhibition of CARM1-Mediated methylation of ACSL4 promotes ferroptosis in colorectal cancer. Adv. Sci. (Weinh).

[44] Quan J., Bode A.M., Luo X. (2021). ACSL family: the regulatory mechanisms and therapeutic implications in cancer. Eur. J. Pharmacol..

[45] Sae-Fung A., Vinayavekhin N., Fadeel B., Jitkaew S. (2024). ACSL3 is an unfavorable prognostic marker in cholangiocarcinoma patients and confers ferroptosis resistance in cholangiocarcinoma cells. NPJ Precis Oncol..

[46] Murphy C.S., Fairfield H., DeMambro V.E., Fadel S., Gartner C.A., Karam M. (2025). Inhibition of acyl-CoA synthetase long-chain isozymes decreases multiple myeloma cell proliferation and causes mitochondrial dysfunction. Mol. Oncol..

[47] Cao Y., Li J., Chen Y., Wang Y., Liu Z., Huang L. (2025). Monounsaturated fatty acids promote cancer radioresistance by inhibiting ferroptosis through ACSL3. Cell Death Dis..

[48] Padanad M.S., Konstantinidou G., Venkateswaran N., Melegari M., Rindhe S., Mitsche M. (2016). Fatty acid oxidation mediated by Acyl-CoA synthetase long chain 3 is required for mutant KRAS lung tumorigenesis. Cell Rep..

[49] Quan J., Cheng C., Tan Y., Jiang N., Liao C., Liao W. (2022). Acyl-CoA synthetase long-chain 3-mediated fatty acid oxidation is required for TGFbeta1-induced epithelial-mesenchymal transition and metastasis of colorectal carcinoma. Int. J. Biol. Sci..

[50] Togashi A., Katagiri T., Ashida S., Fujioka T., Maruyama O., Wakumoto Y. (2005). Hypoxia-inducible protein 2 (HIG2), a novel diagnostic marker for renal cell carcinoma and potential target for molecular therapy. Cancer Res..

[51] Povero D., Johnson S.M., Liu J. (2020). Hypoxia, hypoxia-inducible gene 2 (HIG2)/HILPDA, and intracellular lipolysis in cancer. Cancer Lett..

[52] Tavaglione F., Pennisi G., Pelusi S. (2025). PNPLA3 I148M and hepatocellular carcinoma. Liver Int..

[53] Wijarnpreecha K., Scribani M., Raymond P., Harnois D.M., Keaveny A.P., Ahmed A. (2021). PNPLA3 gene polymorphism and Liver- and extrahepatic cancer-related mortality in the United States. Clin. Gastroenterol. Hepatol..

[54] Cruz A.L.S., Barreto E.A., Fazolini N.P.B., Viola J.P.B., Bozza P.T. (2020). Lipid droplets: platforms with multiple functions in cancer hallmarks. Cell Death Dis..

[55] Heller S., Cable C., Penrose H., Makboul R., Biswas D., Cabe M. (2016). Intestinal inflammation requires FOXO3 and prostaglandin E2 dependent lipogenesis and elevated lipid droplets. Am. J. Physiol. Gastrointest. Liver Physiol..

[56] Lee H., Horbath A., Kondiparthi L., Meena J.K., Lei G., Dasgupta S. (2024). Cell cycle arrest induces lipid droplet formation and confers ferroptosis resistance. Nat. Commun..

[57] Pagliari F., Jansen J., Knoll J., Hanley R., Seco J., Tirinato L. (2024). Cancer radioresistance is characterized by a differential lipid droplet content along the cell cycle. Cell Div..

[58] Liu M., Fang X., Wang H., Ji R., Guo Q., Chen Z. (2022). Characterization of lipid droplet metabolism patterns identified prognosis and tumor microenvironment infiltration in gastric cancer. Front. Oncol..

[59] Ma H., Yang W., Wang X., Dai G. (2022). PRR11 promotes proliferation and migration of colorectal cancer through activating the EGFR/ERK/AKT pathway via increasing CTHRC1. Ann. Clin. Lab. Sci..

[60] Liu L., Jiang H., Zhao W., Meng Y., Li J., Huang T. (2019). Cdc42-mediated supracellular cytoskeleton induced cancer cell migration under low shear stress. Biochem. Biophys. Res. Commun..

[61] Du N., Li D., Zhao W., Liu Y. (2024). Stratifin (SFN) regulates cervical cancer cell proliferation, apoptosis, and cytoskeletal remodeling and metastasis progression through LIMK2/Cofilin signaling. Mol. Biotechnol..

[62] Murata D., Azuma K., Murotani K., Zaizen Y., Hoshino T. (2025). Impact of stratifin/14-3-3sigma on survival and interstitial lung disease in patients with non-small cell lung cancer receiving immune checkpoint inhibitors. Cancer Immunol. Immunother..

[63] Umbricht C.B., Evron E., Gabrielson E., Ferguson A.T., Marks J., Sukumar S. (2025). Correction: hypermethylation of 14-3-3 sigma (stratifin) is an early event in breast cancer. Oncogene.

[64] Ovejero S., Kumanski S., Soulet C., Azarli J., Pardo B., Santt O. (2023). A sterol-PI(4)P exchanger modulates the Tel1/ATM axis of the DNA damage response. EMBO J..

[65] Thapa N., Wen T., Cryns V.L., Anderson R.A. (2023). Regulation of cell adhesion and migration via microtubule cytoskeleton Organization, cell polarity, and phosphoinositide signaling. Biomolecules.

[66] Zembroski A.S., Andolino C., Buhman K.K., Teegarden D. (2021). Proteomic characterization of cytoplasmic lipid droplets in human metastatic breast cancer cells. Front. Oncol..

[67] Kumar Y.P., Muthukrishnan A., Rama Rao G.R., Pavankumar Y.S. (2023). Role of desmosomal components in the initiation and metastasis of oral cancer-a review. J. Oral. Maxillofac. Pathol..

[68] Wang Q., Qi C., Min P., Wang Y., Ye F., Xia T. (2022). MICAL2 contributes to gastric cancer cell migration via Cdc42-dependent activation of E-cadherin/beta-catenin signaling pathway. Cell Commun. Signal..

[69] Chen N., Han X., Bai X., Yin B., Wang Y. (2020). LASP1 induces colorectal cancer proliferation and invasiveness through hippo signaling and Nanog mediated EMT. Am. J. Transl. Res..

[70] Aggelou H., Chadla P., Nikou S., Karteri S., Maroulis I., Kalofonos H.P. (2018). LIMK/cofilin pathway and slingshot are implicated in human colorectal cancer progression and chemoresistance. Virchows Arch..

