# Supplementary material for: Lipid droplet-associated proteins integrate lipid metabolism, cell cycle, and cytoskeletal remodeling in colon cancer
Source: J Lipid Res. 2026 Oct 6;67(11):101165. doi: 10.1016/j.jlr.2026.101165 (PMC13755470; doi:10.1016/j.jlr.2026.101165)

Supplemental Figure 1

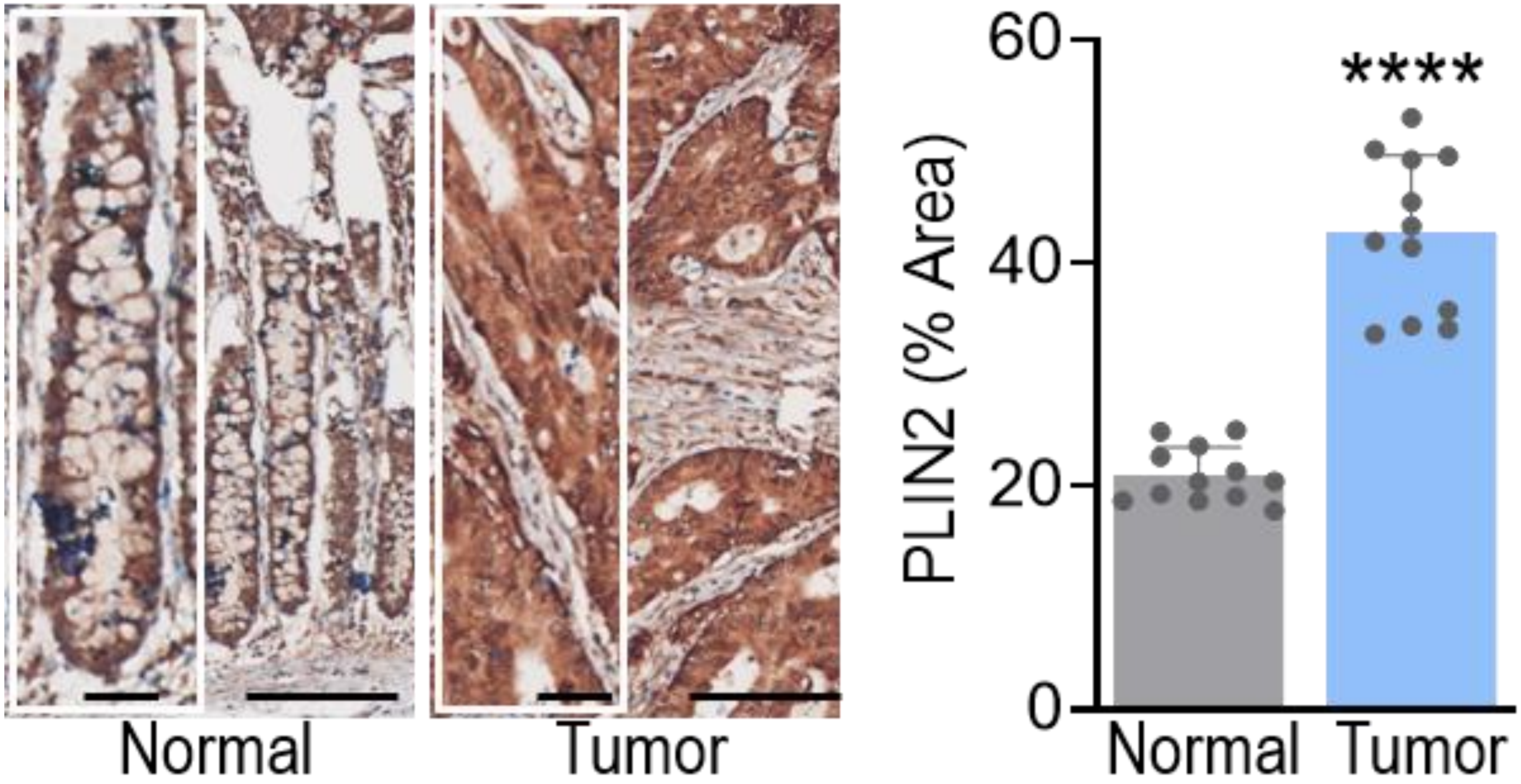

Supplement: Supplementary file 1 [file mmc1.pdf]
