# Supplementary material for: Lipid droplet-associated proteins integrate lipid metabolism, cell cycle, and cytoskeletal remodeling in colon cancer
Source: J Lipid Res. 2026 Oct 6;67(11):101165. doi: 10.1016/j.jlr.2026.101165 (PMC13755470; doi:10.1016/j.jlr.2026.101165)
Supplement: Supplementary file 6 [file mmc6.docx]

**Supplemental Table 1.** **Lipid droplet–associated proteins and their functions (GeneCards).** The lipid droplet transcriptomic signature (LDs_151_) was generated from publicly available lipid droplet proteomic datasets through protein-to-gene mapping and functional annotation.

| **Rank** | **Gene** | **Gene name** | **Function** |
| --- | --- | --- | --- |
| **1** | ARL1 | ARF Like GTPase 1 | Recruits golgins, arfaptins, and Arf-GEFs to the trans-Golgi network, and modulates their functions at the Golgi complex. |
| **2** | C18orf32 | Chromosome 18 Open Reading Frame 32 | Involved in the positive regulation of the NF-kappa-B signaling pathway. |
| **3** | RAB18 | Ras-Related Protein Rab-18 | Localizes ZFYVE1 to lipid droplets and mediates the formation of endoplasmic reticulum-lipid droplet. |
| **4** | GAPDH | Glyceraldehyde-3-Phosphate Dehydrogenase | Promotes cancer metastasis by influencing the process of epithelial-mesenchymal transition. |
| **5** | ACSL4 | Acyl-CoA Synthetase Long Chain Family Member 4 | Catalyzes the conversion of long-chain fatty acids to their active form acyl-CoA for synthesis of cellular lipids and degradation. |
| **6** | LPCAT1 | Lysophosphatidylcholine Acyltransferase 1 | Influences lipid metabolism and plasma membrane remodeling, which creates a cellular environment conducive to cancer cell proliferation and survival. |
| **7** | RAB13 | Ras-Related Protein Rab-13 | Involved in endocytic recycling and regulates the transport to the plasma membrane of transmembrane proteins. |
| **8** | ATG2A | Autophagy Related 2A | Plays a role in autophagosome formation and regulation of LD morphology. |
| **9** | NBAS | NBAS Subunit Of NRZ Tethering Complex | Involved in Golgi-to-endoplasmic reticulum retrograde transport. |
| **10** | EFCAB4B | Calcium Release Activated Channel Regulator 2A | Plays a role in cellular trafficking and calcium signaling. |
| **11** | ABHD5 | Abhydrolase Domain Containing 5, Lysophosphatidic Acid Acyltransferase | Suppresses aerobic glycolysis, promotes AMPK/p53 pathway activation, and inhibits epithelial-mesenchymal transition. |
| **12** | AGPAT9 | 1-Acylglycerol-3-Phosphate O-Acyltransferase 9 | Plays a role in lipid metabolism, and is essential for building cell membranes. |
| **13** | DHRS1 | Dehydrogenase/Reductase (SDR Family) Member 1 | NADPH-dependent oxidoreductase which catalyzes the reduction of steroids. |
| **14** | SNAP29 | Synaptosomal-Associated Protein 29 | Aids in the fusion of autophagosomes with lysosomes, and influences cell migration |
| **15** | PEX13 | Peroxisomal Biogenesis Factor 13 | Involved in lipid metabolism |
| **16** | BAX | BCL2-Associated X, Apoptosis Regulator | Plays a role in the mitochondrial apoptotic process |
| **17** | C7orf68 (HILPDA) | Hypoxia Inducible Lipid Droplet Associated | Increases intracellular lipid accumulation, stimulates expression of cytokines, and enhances cell growth and proliferation. |
| **18** | NSDHL | NAD(P) Dependent 3-Beta-Hydroxysteroid Dehydrogenase NSDHL | Involved in cholesterol biosynthesis. |
| **19** | ARL8A | ARF Like GTPase 8 | Plays a role in lysosome motility. |
| **20** | AIFM2 | Apoptosis-Inducing Factor, Mitochondrion-Associated 2 | Influences tumor promotion and suppression depending on cellular context. |
| **21** | AAAS | Aladin WD Repeat Nucleoporin | Plays a role in the normal development of the peripheral and central nervous system |
| **22** | HSDL1 | Hydroxysteroid Dehydrogenase Like 1 | Plays a role in the metabolism of steroid hormones. |
| **23** | C2orf47 | M-AAA Protease-Interacting Protein 1, Mitochondrial | Facilitates epithelial-mesenchymal transition. |
| **24** | APOOL | Apolipoprotein O Like | Specifically binds to cardiolipin (in vitro), but not to the precursor lipid phosphatidylglycerol. |
| **25** | ELMOD2 | ELMO Domain Containing 2 | Acts as a GTPase-activating protein (GAP) toward guanine nucleotide exchange factors like ARL2, ARL3, Arf1, and ARF6, but not the GTPases outside the Arf family. |
| **26** | CASP14 | Caspase 14 | Plays a role in epithelial differentiation. |
| **27** | HIST1H2AJ | Histone Cluster 1, H2aj | Promotes cell proliferation, regulates differentiation. |
| **28** | RAB3B | RAB3B, Member RAS Oncogene Family | Involved in regulating lipid metabolism and signaling pathways |
| **29** | PNPLA3 | Patatin-Like Phospholipase Domain-Containing Protein 3 | Plays a role in acyl-chain remodeling of triglycerides. |
| **30** | MAGEA4 | Melanoma-Associated Antigen 4 | Regulates cell proliferation through inhibition of cell cycle arrest at the G1 phase and negatively regulates p53-mediated apoptosis. |
| **31** | PRR11 | Proline Rich 11 | Plays a critical role in cell cycle progression. |
| **32** | C17orf80 (MIG3) | Chromosome 17 Open Reading Frame 80 | Plays a critical role in mitochondrial metabolism and cell proliferation. |
| **33** | RAB2B | RAB2B, Member RAS Oncogene Family | Acts as an oncogene, driving tumor initiation through its interactions with pectin and disruption of actin cytoskeleton. |
| **34** | RAB33B | RAB33B, Member RAS Oncogene Family | Involved in autophagy regulation and membrane trafficking. |
| **35** | RAB43 | RAB43, Member RAS Oncogene Family | Involved in retrograde transport from the endocytic pathway to the Golgi apparatus. |
| **36** | HSD17B7 | Hydroxysteroid (17-Beta) Dehydrogenase 7 | Involved in sex steroid and cholesterol biosynthesis. |
| **37** | ZW10 | ZW10 Kinetochore Protein | Involved in cell division, proliferation, and potentially acts as a biomarker for colon cancer. |
| **38** | PKMYT1 | Protein Kinase, Membrane Associated Tyrosine/Threonine 1 | Inhibits activity of CDK1, preventing mitosis. |
| **39** | NDE1 | NudE Neurodevelopment Protein 1 | Regulates cell division and proliferation, influences migration and invasion, modulates apoptosis. |
| **40** | RAB21 | RAB21, Member RAS Oncogene Family | Promotes and suppresses tumor growth depending on cellular context. Also influences endosomal trafficking. |
| **41** | SPRR3 | Small Proline-Rich Protein 3 | Cross-linked envelope protein of keratinocytes. |
| **42** | COPS3 | COP9 Singlaosome Subunit 3 | Activates the MEK/ERK pathway, facilitating cell proliferation, invasion, and metastasis. |
| **43** | NDUFB6 | NADH:Ubiquinone Oxidoreductase Subunit B6 | Involved in mitochondrial dysfunction and radioresistance and mitochondrial metabolic reprogramming. |
| **44** | SELK | Selenoprotein K | May influence tumor development through interaction with the WNT signaling pathway. |
| **45** | RAB32 | RAB32, Member RAS Oncogene Family | Plays a role in colon cancer development, specifically in tumor with microsatellite instability. |
| **46** | C12orf23 | Chromosome 12 Open Reading Frame 23 | May promote or inhibit cancer development depending on cellular context. |
| **47** | FAM114A1 | Family With Sequence Similarity 114 Member A1 | Modulates AKT1 signaling pathway, regulates cell proliferation, epithelial-mesenchymal transition, and migration. |
| **48** | TMEM159 | Transmembrane Protein 159 | Aids in the formation of lipid droplets (LD), and defines the sides of LD formation in the ER. |
| **49** | BAG2 | BAG Cochaperone 2 | Promotes proliferation and chemoresistance in CRC cells. |
| **50** | NDN | Necdin, MAGE Family Member | Acts as a growth suppressor that facilitates the entry of the cell into cell cycle arrest. |
| **51** | SPG20 | Spastic Paraplegia 20 | Binds and transfers all lipid species found in lipid droplets, from phospholipids to triglycerides and sterol esters. |
| **52** | RAB34 | RAB34, Member RAS Oncogene Family | Promotes cell migration and invasion in CRC cells. |
| **53** | RAB12 | RAB12, Member RAS Oncogene Family | Involved in membrane trafficking and regulation of mTORC1 activity and autophagy. |
| **54** | RAB8B | RAB8B, Member RAS Oncogene Family | Involved in vesicular trafficking and Wnt/Beta-catenin pathway regulation. |
| **55** | ZFYVE1 | Zinc Finger FYVE-Type Containing 1 | Aids in the formation of LDs, regulates the morphology, size and distribution of LDs, mediates formation of endoplasmic reticulum-lipid droplets by forming a complex with RAB18 and ZW10. |
| **56** | USP33 | Ubiquitin Specific Peptidase 33 | Inhibits cell migration through the Slit-Robo signaling pathway, promotes metastasis by regulating C-Met proteins and affecting beta-arrestin2-dependent signaling. |
| **57** | BAG5 | BAG Cochaperone 5 | Stabilizes mutp53, promoting cancer cell proliferation, tumor growth, and metastasis. |
| **58** | MAPK1IP1L | Mitogen-Activated Protein Kinase 1 Interacting Protein 1 Like | Influences MAPK pathway, regulating cell proliferation, differentiation, and apoptosis. |
| **59** | FAM177A1 | Family With Sequence Similarity 177 Member A1 | May inhibit cell proliferation, while promoting apoptosis and differentiation. |
| **60** | METTL7B | Methyltransferase-Like Protein 7B | Accelerates cancer cell proliferation, contributes to tumor development and metastasis. |
| **61** | RDH11 | Retinol Dehydrogenase 11 | Responsible for cell differentiation and proliferation. |
| **62** | SCCPDH | Saccharopine Dehydrogenase (Putative) | Involved in the glycolipid biosynthetic process and cell cycle regulation. |
| **63** | CLPTM1L | CLPTM1 Regulator of GABA Type A Receptor Forward Trafficking | Plays a role in cell survival signaling and resistance to chemotherapy. |
| **64** | PLIN2 | Perilipin 2 | Structural component of LDs |
| **65** | H1FX | H1 Histone Family, Member X | Responsible for the condensation of nucleosome chains into higher-order structures. |
| **66** | PNPLA2 | Patatin Like Domain 2, Triaglycerol Lipase | Inhibits LDAH-stimulated LD fusion, promotes the breakdown of triglycerides. |
| **67** | RAB11B | RAB11B, Member RAS Oncogene Family | Regulates E-cadherin expression, promotes cell transformation, and promotes cell migration. |
| **68** | PLIN3 | Perilipin 3 | Structural component of LDs. |
| **69** | DHDDS | Dehydrodolichyl Diphosphate Synthase Subunit | Plays a role in the synthesis of dolichol. |
| **70** | RAB27A | RAB27A, Member RAS Oncogene Family | Facilitates the secretion of exosomes, acting as a regulator in cancer progression. |
| **71** | RHOG | Ras Homolog Family Member G | Plays a role in cell migration, proliferation, and survival. |
| **72** | RAB8A | RAB8A, Member RAS Oncogene Family | Progresses tumors when downregulated, involved in Wnt signaling. |
| **73** | ACSL3 | Acyl-CoA Synthetase Long Chain Family Member 3 | Involved in metabolic programming, fatty acid metabolism, epithelial-mesenchymal transition, and ferroptosis resistance. |
| **74** | ACACA | Acetyl-CoA Carboxylase Alpha | Increases cell growth and proliferation when overexpressed, influences fatty acid synthesis and growth. |
| **75** | LSS | Lanosterol Synthase | Suppresses cancer progression by influencing the immune microenvironment. |
| **76** | FAR1 | Fatty Acyl-CoA Reductase 1 | Plays a role in the production of ether lipids/plasmalogens which synthesis requires fatty alcohols. |
| **77** | OPA1 | OPA1 Mitochondrial Dynamin Like GTPase | Maintains mitochondrial structure and function, increasing cell survival and proliferation. |
| **78** | KIF16B | Kinesin Family Member 16B | Involved in the remodeling of the tumor microenvironment, promoting the spread and progression of colon cancer. |
| **79** | FAF2 | Fas Associated Factor Family Member | Promotes expression of molecules involved in cell adhesion and extravasation. |
| **80** | RAB5A | RAB5A, Member RAS Oncogene Family | Promotes cell motility and invasion, influences integrin-mediated signaling and the formation of invadosomes. |
| **81** | TBC1D5 | TBC1 Domain Family Member 5 | Acts as a molecular switch between endosomal and autophagosomal transport and reprogramming vesicle trafficking upon autophagy induction. |
| **82** | RAB3GAP1 | RAB3 GTPase Activating Protein Catalytic Subunit 1 | Potential tumor suppressor, acting as a molecular switch in vesicle trafficking and autophagy. |
| **83** | VPS13C | Vacuolar Protein Sorting 13 Homolog C | Mediates the transfer of lipids between membranes at organelle contact sites. |
| **84** | ZC3HAV1 | Zinc Finger CCCH-Type Containing, Antiviral 1 | Acts as a tumor suppressor when downregulated by inhibiting cancer cell growth and promoting apoptosis. |
| **85** | AGPAT6 | Glycerol-3-Phosphate Acyltransferase 4 | Protects against lipotoxicity. |
| **86** | RAB2A | RAB2A, Member RAS Oncogene Family | Influences cellular processes like proliferation, migration, and invasion. |
| **87** | RAB15 | RAB15, Member RAS Oncogene Family | Involved in metabolic activity, cell cycle, and vesicular trafficking. |
| **88** | NUS1 | NUS1 Dehydrolichyl Diphosphate Synthase Subunit | Elevated expression in colon cancer, leading to cell proliferation, migration, and invasion. |
| **89** | PITPNB | Phosphatidylinositol Transfer Protein Beta | Plays a role in membrane trafficking |
| **90** | UBE2G2 | Ubiquitin Conjugating Enzyme E2 G2 | Aids in the transfer of ubiquitin, may act as a tumor suppressor or promoter depending on cellular context. |
| **91** | DSP | Desmoplakin | Inhibits the Wnt/B-catenin signaling pathway. |
| **92** | JUP | Junction Plakoglobin | A key component of cell-cell adhesion structures, involved in the Wnt/Beta-catenin signaling pathway. |
| **93** | LASP1 | LIM And SH3 Protein 1 | Facilitates the epithelial-mesenchymal transition, promoting tumor cell proliferation and invasiveness. |
| **94** | ACTG1 | Actin Gamma 1 | A key component of the actin cytoskeleton contributes to tumor growth and spread. |
| **95** | MICAL2 | Microtubule Associated Monooxygenase, Calponin, And LIM Domain Containing 2 | Promotes cancer cell growth and migration, influences the Wnt/Beta-catenin signaling pathway, interacts with and degrades p53. |
| **96** | RAB5C | RAB5C, Member RAS Oncogene Family | Promotes radio resistance in rectal adenocarcinoma and contributes to chemotherapy resistance. |
| **97** | CYB5R3 | Cytochrome B5 Reductase 3 | Acts as a tumor suppressor by inducing apoptosis and inhibiting tumor growth, it can also promote metastasis. |
| **98** | VIM | Vimentin | Involved in epithelial-mesenchymal transition, aiding in cancer cell migration and invasion. |
| **99** | VAT1 | Vesicle Amine Transport 1 | Involved in the trafficking of amin-containing vesicles and maintaining proper cellular function. |
| **100** | IL32 | Interleukin | Plays a role in innate and adaptive immune responses. |
| **101** | LDHA | Lactate Dehydrogenase A | Elevated LDHA expression leads to increased glycolysis which supports cancer cell growth and proliferation. |
| **102** | ARFGAP1 | ARF GTPase Activating Protein 1 | GTPase-activating protein for ARF1, which is involved in membrane trafficking and actin remodeling. |
| **103** | ARF5 | ARF GTPase 5 | GTP-binding protein involved in protein trafficking, modulates vesicle budding and uncoating within the Golgi apparatus. Plays a role in cell proliferation, motility, and differentiation by regulating membrane traffic and cytoskeleton association. |
| **104** | AUP1 | AUP1 Lipid Droplet Regulating VLDL Assembly Factor | Plays a role in lipid droplet formation, induces lipid droplet clustering. |
| **105** | RALA | RAS Like Proto-Oncogene A | Involved in gene expression, cell migration, cell proliferation, oncogenic transformation, and membrane trafficking. |
| **106** | YKT6 | YKT6 V-SNARE Homolog | Functions in endoplasmic reticulum to Golgi transport. |
| **107** | WIPI2 | WD Repeat Domain, Phosphoinositide Interacting 2 | Involved in autophagosomes, leading to autophagy and promotion of tumor growth |
| **108** | HSPA8 | Heat Shock Protein Family A (Hsp70) Member 8 | Interacts with and degrades CAV1, leading to the nuclear translocation of beta-catenin. |
| **109** | HSP90AB1 | Heat Shock Protein 90 Alpha Family Class B Member 1 | Ensures proteins fold correctly and maintain stability, promotes tumor cell survival, and inhibits apoptosis. |
| **110** | TUBB | Tubulin Beta Class I | A protein coding gene, related pathways include loss of proteins required for interphase microtubule organization from the centrosome and cell cycle. |
| **111** | VCP | Valosin Containing Protein | Plays a role in tumor growth by activating STAT3 signaling and increasing fatty acid oxidation. |
| **112** | PRKDC | Protein Kinase, DNA-Activated, Catalytic Subunit | Involved in DNA double-strand break repair. |
| **113** | SQLE | Squalene Epoxidase | Involved in lipid droplet formation, regulation of cell population proliferation, and sterol biosynthetic process. |
| **114** | YWHAZ | Tyrosine 3-Monooxygenase/Tryptophan 5-Monooxygenase Activation Protein Zeta | Promotes cancer development and progression through roles in cell cycle. |
| **115** | ALDOA | Aldolase, Fructose-Biphosphate A | Plays a key role in glycolysis and gluconeogenesis. |
| **116** | CFL1 | Cofilin 1 | Plays a role in the regulation of cell morphology and cytoskeletal organization in epithelial cells. |
| **117** | RAB1B | RAB1B, Member RAS Oncogene Family | Key regulator of intracellular membrane trafficking and regulates transport between the ER and successive Golgi compartments. |
| **118** | ANXA7 | Annexin A7 | Increases proliferation, migration, and invasion of CRC cells. |
| **119** | ARF4 | ARF GTPase 4 | Acts as both a tumor suppressor and oncogene depending on p53 status and other cellular context. |
| **120** | RHOA | Ras Homolog Family Member A | Binds to a variety of effector proteins to regulate cellular responses such as cytoskeletal dynamics, cell migration, and cell cycle. |
| **121** | RAB14 | RAB14, Member RAS Oncogene Family | Plays a role in receptor trafficking. |
| **122** | CALM1 | Calmodulin 1 | A calcium-binding protein that acts as a critical regulator of cell cycle control, proliferation, and migration. |
| **123** | CAV1 | Caveolin 1 | Encodes for the protein Caveolin-1, and acts as a tumor suppressor and promotor depending on cellular context. |
| **124** | METTL7A | Thiol Methyltransferase 1A | Encodes an RNA methyltransferase and is involved in lipid metabolism and methylation. |
| **125** | RAB5B | RAB5B, Member RAS Oncogene Family | Affects cell signaling and metabolic regulation, and plays a critical role in the production of exosomes in CRC cells. |
| **126** | CDC42 | Cell Division Cycle 42 | Mediates the occurrence of multiple malignant tumors through different pathways. |
| **127** | RAP1B | RAP1B, Member of RAS Oncogene Family | Regulates cell adhesion, growth, and differentiation. |
| **128** | HTATIP2  (TIP30) | HIV-1 Tat Interactive Protein 2 | Plays a role in suppressing tumor growth, promoting apoptosis, and inhibiting metastasis. |
| **129** | NRAS | NRAS Proto-Oncogene, GTPase | Aids in regulating cell growth, division, and survival. |
| **130** | MLX | MAX Dimerization Protein MLX | Plays a role in transcriptional activation of glycolytic target genes and metabolic pathways. |
| **131** | HSD17B11 | Hydroxysteroid 17-Beta Dehydrogenase 11 | Acts as a competing endogenous RNA (ceRNA) by acting as a sponge for miR-388-3p, which is downregulated in CRC cells. |
| **132** | SRI | Sorcin | Enhances metastasis and promotes epithelial-mesenchymal transition. |
| **133** | SLC25A5 | Solute Carrier Family 25 Member 5 | Represses cell growth by inhibiting the MAPK signaling pathway. |
| **134** | RAB6A | RAB6A, Member RAS Oncogene Family | Maintains cancer stem cell phenotypes and regulates cell migration. |
| **135** | RAB7A | RAB7A, Member RAS Oncogene Family | Regulates vesicular trafficking, and acts as a tumor suppressor |
| **136** | RAB10 | RAB10, Member RAS Oncogene Family | Regulates intracellular membrane trafficking and activates the NF-κB signaling pathway. |
| **137** | RAB1A | RAB1A, Member RAS Oncogene Family | Promotes tumor growth and metastasis by activating the mTORC1 signaling pathway and influences cell proliferation. |
| **138** | YWHAQ | Tyrosine 3-Monooxygenase/Tryptophan 5-Monooxygenase Activation Protein Theta | Meditates tumor-promoting functions by activiating the Wnt/Beta-catenin signaling pathway. |
| **139** | UBXN4 | UBX Domain Protein 4 | Activates the Wnt/Beta-catenin signaling pathway, leading to cell proliferation, migration, and invasion. |
| **140** | ANXA1 | Annexin A1 | Acts as a promoter and inhibitor of tumor progression. |
| **141** | ANXA2 | Annexin A2 | Influences the extracellular matrix, leading to tumor cell invasion and metastasis. |
| **142** | LYZ | Lysozyme | Contributes to the formation of cancer stem cell niches, possibly promoting metastasis. |
| **143** | CSTB | Cystatin B | Plays a role in cell proliferation, migration, and metastasis. |
| **144** | SFN | Stratifin | Acts as a tumor suppressor and an oncogene. |
| **145** | PRDX1 | Peroxiredoxin 1 | Participates in the signaling cascades of growth factors and tumor necrosis factor-alpha by regulating the intracellular concentrations of Hydrogen Peroxide. |
| **146** | TXN | Thioredoxin | Plays a role in maintaining redox homeostasis. |
| **147** | RPL23A | Ribosomal Protein L23a | Regulates cell cycle progression in CRC cells, affecting cell division and growth. |
| **148** | UBA52 | Ubiquitin A-52 Residue Ribosomal Protein Fusion Product 1 | Delivers RPL40 and ubiquitin simultaneously to the ribosome. Overexpression leads to increased cell proliferation and migration. |
| **149** | UQCRQ | Ubiquinol-Cytochrome C Reductase Complex III Subunit VII | Part of the mitochondrial electron transport chain, when mutated, can lead to mitochondrial disorders. |
| **150** | TAGLN2 | Transgelin 2 | Promotes cell proliferation, invasion, migration, and epithelial-mesenchymal transition by activating STAT3 signaling pathway. |
| **151** | TUBA1C | Tubulin Alpha 1c | Involved in cell cycle regulation and tumor cell proliferation. |
